# Thiazide Dose, Urine Calcium, and Symptomatic Kidney Stone Events

**DOI:** 10.1001/jamanetworkopen.2024.28953

**Published:** 2024-08-22

**Authors:** Ryan S. Hsi, Phyllis L. Yan, Naim M. Maalouf, Sara L. Best, John R. Asplin, Vahakn Shahinian, John M. Hollingsworth

**Affiliations:** 1 Department of Urology, Vanderbilt University Medical Center, Nashville, Tennessee; 2 Dow Division of Health Services Research, Department of Urology, University of Michigan, Ann Arbor; 3 The Charles and Jane Pak Center for Mineral Metabolism and Clinical Research, University of Texas Southwestern Medical Center, Dallas; 4 Department of Internal Medicine, University of Texas Southwestern Medical Center, Dallas; 5 Department of Urology, University of Wisconsin–Madison; 6 Labcorp, Itasca, Illinois; 7 Division of Nephrology, Department of Internal Medicine, University of Michigan, Ann Arbor; 8 Department of Urology, NorthShore University Health System, Evanston, Illinois

## Abstract

This cohort study examines the association between thiazide dose and urine calcium reduction and correlates urine calcium changes with the occurrence of symptomatic kidney stone events.

## Introduction

Although thiazides are a mainstay of kidney stone prevention,^1^ one study (the Hydrochlorothiazide for Kidney Stone Recurrence Prevention [NOSTONE] trial) questioned their efficacy,^2^ leading some to argue against their use for this indication.^3^ Because thiazides are believed to mitigate stone formation by reducing urine calcium, one explanation for NOSTONE’s null finding is that the calcium reductions were insufficient to affect recurrence risk. We conducted a cohort study quantifying the association between thiazide dose and calcium reduction and correlating calcium changes with symptomatic stone events.

## Methods

We analyzed the Medicare-Litholink Database,^4^ identifying adults with kidney stones who underwent initial 24-hour urine collection for stone risk assessment between January 1, 2011, and December 31, 2018 (eMethods, eFigure 1, and eTables 1 and 2 in Supplement 1). We identified those prescribed thiazides (hydrochlorothiazide, chlorthalidone, or indapamide) within the 6 months after urine collection and those who also had follow-up collection between 30 and 180 days after their first prescription fill. The University of Michigan Health System institutional review board deemed this study exempt and did not require consent because they considered the study to be no more than minimal risk. This study followed the STROBE reporting guideline.

**Figure 1.**
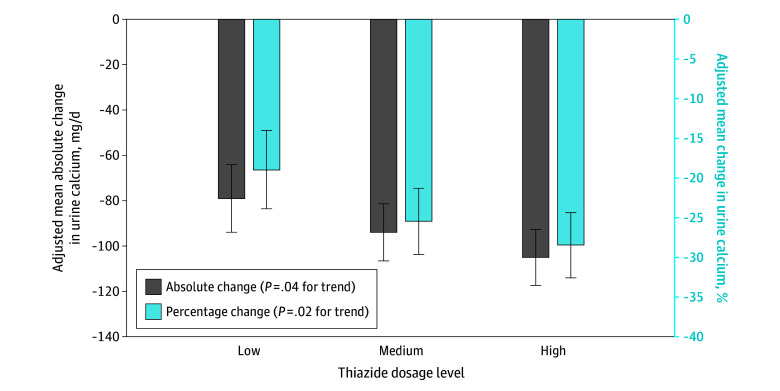
Adjusted Mean Absolute and Percentage Change in Urine Calcium by Daily Thiazide Dose Doses are classified as low (<12.5 mg/d chlorthalidone, <0.6125 mg/d indapamide, <25 mg/d hydrochlorothiazide), medium (12.5 to <25 mg/d chlorthalidone, 0.6125 to <1.25 mg/d indapamide, 25 to <50 mg/d hydrochlorothiazide), and high (≥25 mg/d chlorthalidone, ≥1.25 mg/d indapamide, ≥50 mg/d hydrochlorothiazide). Error bars indicate 95% CIs.

We evaluated changes in calcium based on low, medium, and high thiazide dosages (Figure 1). We fit multivariable linear regression models to adjust for age, sex, race and ethnicity, dual Medicare-Medicaid eligibility status, region of residence, level of comorbid illness, whether the participant had conditions increasing recurrence risk,^5^ medication adherence,^6^ and baseline urine calcium level.

We assigned participants into terciles based on the magnitude of the calcium change. We used the Kaplan-Meier method and multivariable Cox proportional hazards regression modeling to compare the incidence of a symptomatic stone event, defined as emergency department visit, hospitalization, or surgery for stones. We assessed for the first occurrence of an event 6 to 48 months after the initial prescription fill. We adjusted for the same variables, except for medication adherence and baseline urine calcium level. Analyses were conducted with SAS software. Tests were 2-sided, and *P* < .05 was considered statistically significant.

## Results

**Figure 2.**
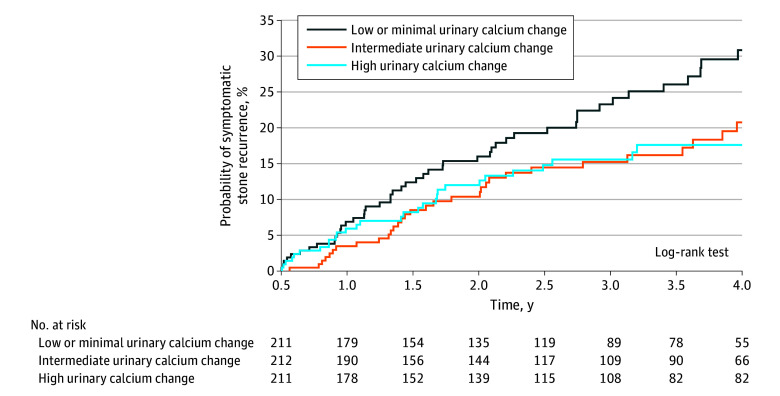
Kaplan-Meier Curves of Cumulative Incidence of a Symptomatic Stone Event at 4 Years of Follow-Up Across Individuals by Terciles With low or minimal (mean [SD], 24 [61] mg/d), intermediate (mean [SD], −90 [25] mg/d), and high (mean [SD], −216 [74] mg/d) reductions in urine calcium.

The study included 634 participants (mean [SD] age, 67.6 [8.6] years; 339 men [53.5%] and 295 women [46.5%]; 2 Asian [0.3%], 12 Black [1.9%], 2 Hispanic [0.3%], 2 North American Native [0.3%], 602 White [95.0%], 2 of other races and ethnicities [0.3%], and 12 of unknown race and ethnicity [1.9%]). Figure 1 reveals significant associations between higher thiazide doses and greater 24-hour mean (SE) and percentage calcium reductions (low dose: −79.3 [7.6] mg/d [95% CI, −94.1 to −64.4 mg/d] and −18.9% [2.5%] [95% CI, −23.9% to −14.0%]; medium dose: −94.1 [6.4] mg/d [95% CI, −106.7 to −81.5 mg/d] and −25.5% [2.1%] [95% CI, −29.6% to −21.3%]; high dose: −104.6 [6.3] mg/d [95% CI, −117.0 to −92.3 mg/d] and −28.4% [2.1%] [95% CI, −32.6% to −24.4%]; *P* = .04 for absolute difference and *P* = .02 for percentage difference). The adjusted cumulative incidence of a symptomatic stone event at 4 years was 28.8% (95% CI, 21.1%-35.7%), 19.5% (95% CI, 12.9%-25.7%), and 18.0% (95% CI, 11.5%-24.0%) for patients in the low (mean [SD], 24 [61] mg/d), medium (mean [SD], −90 [25] mg/d), and high (mean [SD], −216 [74] mg/d) tercile, respectively, of calcium change after thiazide prescription (*P* = .04 for trend; Figure 2).

## Discussion

Increasing thiazide dosage was associated with greater reductions in calcium, which was negatively correlated with symptomatic stone events. The calcium reductions observed were greater than those in NOSTONE (−42 to −51 mg/d),^2^ which may explain NOSTONE’s null results. Limitations include the possibility of omitted variable bias and unmeasured differences between participants prescribed different dosages that could confound the observed associations. We analyzed a sample of mostly older adults, potentially limiting generalizability. Nonetheless, for individuals prescribed thiazides for stone prevention, it may be beneficial to monitor calcium excretion and adjust dose and diet to attain an adequate reduction in urine calcium.

## Notes

Supplement 1.
eMethods.
**eFigure.** Cohort Flow Diagram **eTable 1.** National Drug Codes to Identify Prescription Fills for a Thiazide Agent Code **eTable 2.** Algorithm for Identifying High-Risk Kidney Stone Patients

Supplement 2.
Data Sharing Statement
