# Supplementary material for: Thiazide Dose, Urine Calcium, and Symptomatic Kidney Stone Events
Source: JAMA Netw Open. 2024 Aug 22;7(8):e2428953. doi: 10.1001/jamanetworkopen.2024.28953 (PMC11342138; doi:10.1001/jamanetworkopen.2024.28953)
Supplement: Supplement 1. — eMethods. eFigure. Cohort Flow Diagram eTable 1. National Drug Codes to Identify Prescription Fills for a Thiazide Agent Code eTable 2. Algorithm for Identifying High-Risk Kidney Stone Patients [file jamanetwopen-e2428953-s001.pdf]

Supplementary Online Content

Hsi RS, Yan PL, Maalouf NM, et al. Thiazide dose, urine calcium, and symptomatic kidney stone events. *JAMA Netw Open.* 2024;7(8):e2428953. doi:10.1001/jamanetworkopen.2024.28953

eMethods.
eFigure. Cohort Flow Diagram
eTable 1. National Drug Codes to Identify Prescription Fills for a Thiazide Agent Code
eTable 2. Algorithm for Identifying High-Risk Kidney Stone Patients

This supplementary material has been provided by the authors to give readers additional information about their work.

© 2024 Hsi RS et al. *JAMA Network Open.*

eMethods.

Study population

We identified Medicare beneficiaries who had a 24-hour urine collection (Litholink, Labcorp, Itasca, IL) between January 1, 2011 and December 31, 2018. We used National Drug Codes (see eTable 1 below) to identify Medicare beneficiaries that had a prescription fill for a thiazide agent, specifically hydrochlorothiazide (HCTZ), chlorthalidone, or indapamide, within six months following their 24-hour urine collection. We identified the subset of beneficiaries who had a follow-up collection between 30 and 180 days after their first prescription fill of the thiazide agent. To allow for comorbidity adjustment and adequate follow up, we excluded beneficiaries without continuous enrollment in Medicare Parts A and B during the 1 year prior to the baseline 24- hour urine through 6 months after the initial prescription. To ensure capture of prescription coverage, we excluded beneficiaries without continuous enrollment in Medicare Part D during the 6 months prior to the baseline 24-hour urine through 4 months after the initial prescription to assess for adherence. We excluded those receiving thiazides or alkali citrate up to 6 months prior to the baseline 24-hour urine. We further excluded those receiving alkali citrate or allopurinol up to 120 days after thiazide initiation, those who changed daily dosing of thiazide between the baseline and follow-up urine collections, and those who had either inadequate baseline or follow-up 24-hour urine collections (normal 24-hour creatinine/kilogram body weight 11.9-24.4 mg/kg males, 8.7 to 20.3 mg/kg females). See the cohort flow diagram in eFigure 1 below for more details.

Outcome assessment

To determine the urine calcium change, we calculated the difference between the urine calcium levels measured at the baseline 24-hour urine collection and the follow-up 24-hour urine collection.

To evaluate for symptomatic stone events, we identified the first occurrence of any stone-related emergency department visit, hospitalization, or surgery between six months and 4 years after the initial thiazide prescription. We identified emergency department visits by the presence of outpatient claims with revenue center codes 0450 to 0459 or 0981 or a non-zero emergency department charge amount in the MedPAR file (https://www.resdac.org/articles/how-identify-hospital-claims-emergency-room-visits-medicare-claims-data). We identified hospitalizations

using claims in the MedPAR file and surgery from ICD procedure and CPT codes (see below for codes). We defined emergency department visits and hospitalizations as stone related if they had an associated primary diagnosis of kidney stone disease during the encounter (see below for codes).

Covariates

We assessed for the following covariates including age at first 24-hour urine test, sex, race/ethnicity, level of comorbid illness (number of hierarchical condition categories), region of residence, dual Medicare-Medicaid eligibility status, high-risk status for stone recurrence, medication adherence, and baseline urine calcium at first 24-hour urine test. Level of comorbid illness was measured using diagnoses from the year prior to the first 24-hour urine test (PMID: 15493448). Medication adherence was defined as $\geq$80% days covered from start of thiazide prescription fill to 6 months (PMID: 26485048). High-risk status was determined if there were diagnoses putting the patient at higher risk for kidney stones in the year prior to the first 24-hour urine test (PMID: 35182586, see eTable 2).

Statistical Analysis

We compared adjusted mean absolute and mean percent differences of urinary calcium for low, medium, and high thiazide daily doses. Doses were classified as low (chlorthalidone <12.5mg per day, indapamide <0.6125mg per day, HCTZ <25mg per day), medium (chlorthalidone 12.5mg to <25mg per day, indapamide 0.6125 to <1.25mg per day, HCTZ 25mg to <50mg per day), and high (chlorthalidone 25mg or higher, indapamide 1.25mg per day or higher, HCTZ 50mg per day or higher). We then evaluated changes in urine calcium based on the dosage prescribed, adjusting for age at first 24-hour urine test, sex, race/ethnicity, level of comorbid illness (number of hierarchical condition categories), region of residence, dual Medicare-Medicaid eligibility status, high-risk status for stone recurrence, medication adherence, and baseline urine calcium at first 24-hour urine test. For these comparisons, we used linear regression models.

To assess stone-related clinical events after thiazide initiation related to urine calcium changes, we categorized individuals from the entire cohort into terciles of urine calcium changes between the first and follow-up 24 hour urine test by absolute change. We used the Kaplan-Meier method

© 2024 Hsi RS et al. *JAMA Network Open.*

to evaluate for an association between these terciles and the unadjusted cumulative incidence of a composite clinical stone event. We used the log-rank test to compare differences in the cumulative incidence of a composite clinical stone events. Then, we performed multivariable Cox modeling to compare the incidence of a symptomatic stone event with respect to the tercile categories. We adjusted for age at first 24-hour urine test, sex, race/ethnicity, level of comorbid illness (number of hierarchical condition categories), region of residence, dual Medicare-Medicaid eligibility status, and high-risk status for stone recurrence. We did not adjust for medication adherence or baseline urine calcium, since they are highly collinear with changes in urine calcium. We assessed for the occurrence of an event beginning six months after each beneficiary's first prescription fill, up to 4 years after the prescription fill. We censored participants at death or if they lost their health insurance coverage.

We conducted all analyses using SAS software, Version 9.4 (SAS Institute Inc., Cary, NC). We performed two-sided significance testing with alpha set at 0.05. The Institutional Review Board at the University of Michigan Health System deemed that this study was exempt from its oversight.

© 2024 Hsi RS et al. *JAMA Network Open.*

eFigure. Cohort Flow Diagram

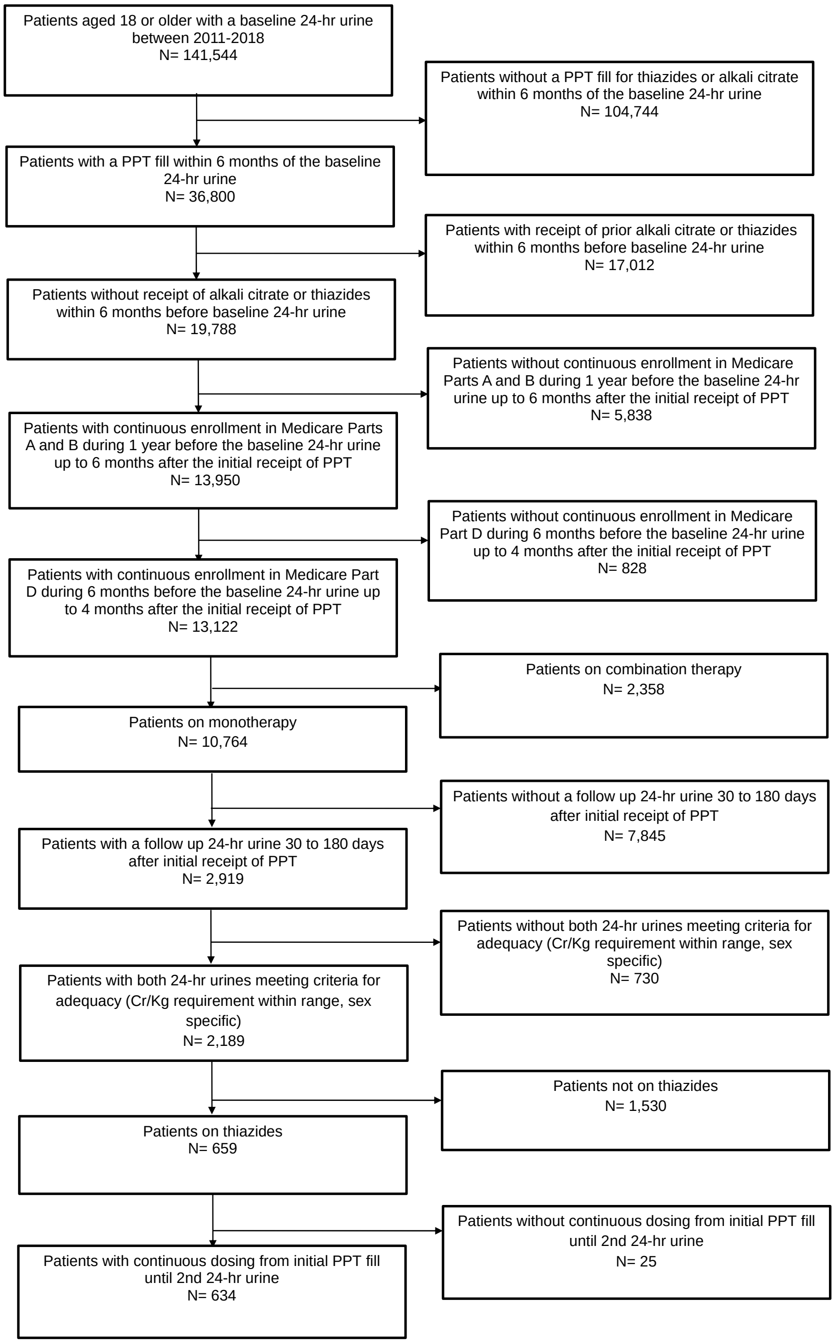

PPT – Preventive pharmacologic therapy

© 2024 Hsi RS et al. *JAMA Network Open.*

eTable 1. National Drug Codes to Identify Prescription Fills for a Thiazide Agent Code

	GNN	Drug_Class
00078061415	ALISKIREN/AMLODIPINE/HCTZ	THIAZIDES
00078052115	ALISKIREN/HYDROCHLOROTHIAZIDE	THIAZIDES
00078052215	ALISKIREN/HYDROCHLOROTHIAZIDE	THIAZIDES
00078052315	ALISKIREN/HYDROCHLOROTHIAZIDE	THIAZIDES
00078052415	ALISKIREN/HYDROCHLOROTHIAZIDE	THIAZIDES
70839011230	ALISKIREN/HYDROCHLOROTHIAZIDE	THIAZIDES
70839012530	ALISKIREN/HYDROCHLOROTHIAZIDE	THIAZIDES
70839031230	ALISKIREN/HYDROCHLOROTHIAZIDE	THIAZIDES
70839032530	ALISKIREN/HYDROCHLOROTHIAZIDE	THIAZIDES
00378057701	AMILORIDE/HYDROCHLOROTHIAZIDE	THIAZIDES
00378057705	AMILORIDE/HYDROCHLOROTHIAZIDE	THIAZIDES
00555048302	AMILORIDE/HYDROCHLOROTHIAZIDE	THIAZIDES
00555048305	AMILORIDE/HYDROCHLOROTHIAZIDE	THIAZIDES
00078055915	AMLODIPINE/VALSARTAN/HCTZ	THIAZIDES
00078056015	AMLODIPINE/VALSARTAN/HCTZ	THIAZIDES
00078056115	AMLODIPINE/VALSARTAN/HCTZ	THIAZIDES
00078056215	AMLODIPINE/VALSARTAN/HCTZ	THIAZIDES
00078056315	AMLODIPINE/VALSARTAN/HCTZ	THIAZIDES
00310011510	ATENOLOL/CHLORTHALIDONE	THIAZIDES
00378206301	ATENOLOL/CHLORTHALIDONE	THIAZIDES
00378206401	ATENOLOL/CHLORTHALIDONE	THIAZIDES
00378206493	ATENOLOL/CHLORTHALIDONE	THIAZIDES
00591578201	ATENOLOL/CHLORTHALIDONE	THIAZIDES
00591578301	ATENOLOL/CHLORTHALIDONE	THIAZIDES
00904620060	ATENOLOL/CHLORTHALIDONE	THIAZIDES
53489053101	ATENOLOL/CHLORTHALIDONE	THIAZIDES
60631041230	AZILSARTAN MED/CHLORTHALIDONE	THIAZIDES
60631042530	AZILSARTAN MED/CHLORTHALIDONE	THIAZIDES
64764094430	AZILSARTAN MED/CHLORTHALIDONE	THIAZIDES
64764099430	AZILSARTAN MED/CHLORTHALIDONE	THIAZIDES
00078045205	BENAZEPRIL/HYDROCHLOROTHIAZIDE	THIAZIDES
00078045405	BENAZEPRIL/HYDROCHLOROTHIAZIDE	THIAZIDES
00172536160	BENAZEPRIL/HYDROCHLOROTHIAZIDE	THIAZIDES
00172536260	BENAZEPRIL/HYDROCHLOROTHIAZIDE	THIAZIDES
00172536360	BENAZEPRIL/HYDROCHLOROTHIAZIDE	THIAZIDES
00185012401	BENAZEPRIL/HYDROCHLOROTHIAZIDE	THIAZIDES
00185020401	BENAZEPRIL/HYDROCHLOROTHIAZIDE	THIAZIDES
00185021101	BENAZEPRIL/HYDROCHLOROTHIAZIDE	THIAZIDES

© 2024 Hsi RS et al. *JAMA Network Open.*

	GNN	Drug_Class
00185023601	BENAZEPRIL/HYDROCHLOROTHIAZIDE	THIAZIDES
00185027701	BENAZEPRIL/HYDROCHLOROTHIAZIDE	THIAZIDES
00185032501	BENAZEPRIL/HYDROCHLOROTHIAZIDE	THIAZIDES
00378472501	BENAZEPRIL/HYDROCHLOROTHIAZIDE	THIAZIDES
00378473501	BENAZEPRIL/HYDROCHLOROTHIAZIDE	THIAZIDES
00378474501	BENAZEPRIL/HYDROCHLOROTHIAZIDE	THIAZIDES
00378477501	BENAZEPRIL/HYDROCHLOROTHIAZIDE	THIAZIDES
00781513101	BENAZEPRIL/HYDROCHLOROTHIAZIDE	THIAZIDES
00781513201	BENAZEPRIL/HYDROCHLOROTHIAZIDE	THIAZIDES
00832048311	BENAZEPRIL/HYDROCHLOROTHIAZIDE	THIAZIDES
00832048411	BENAZEPRIL/HYDROCHLOROTHIAZIDE	THIAZIDES
00832048511	BENAZEPRIL/HYDROCHLOROTHIAZIDE	THIAZIDES
60505026101	BENAZEPRIL/HYDROCHLOROTHIAZIDE	THIAZIDES
60505026201	BENAZEPRIL/HYDROCHLOROTHIAZIDE	THIAZIDES
60505026301	BENAZEPRIL/HYDROCHLOROTHIAZIDE	THIAZIDES
60505026401	BENAZEPRIL/HYDROCHLOROTHIAZIDE	THIAZIDES
64980019401	BENAZEPRIL/HYDROCHLOROTHIAZIDE	THIAZIDES
64980019501	BENAZEPRIL/HYDROCHLOROTHIAZIDE	THIAZIDES
64980019601	BENAZEPRIL/HYDROCHLOROTHIAZIDE	THIAZIDES
00185070101	BISOPROL/HYDROCHLOROTHIAZIDE	THIAZIDES
00185070401	BISOPROL/HYDROCHLOROTHIAZIDE	THIAZIDES
00185070430	BISOPROL/HYDROCHLOROTHIAZIDE	THIAZIDES
00185070701	BISOPROL/HYDROCHLOROTHIAZIDE	THIAZIDES
00185070705	BISOPROL/HYDROCHLOROTHIAZIDE	THIAZIDES
00185070730	BISOPROL/HYDROCHLOROTHIAZIDE	THIAZIDES
00378050101	BISOPROL/HYDROCHLOROTHIAZIDE	THIAZIDES
00378050110	BISOPROL/HYDROCHLOROTHIAZIDE	THIAZIDES
00378050301	BISOPROL/HYDROCHLOROTHIAZIDE	THIAZIDES
00378050310	BISOPROL/HYDROCHLOROTHIAZIDE	THIAZIDES
00378050501	BISOPROL/HYDROCHLOROTHIAZIDE	THIAZIDES
00378050505	BISOPROL/HYDROCHLOROTHIAZIDE	THIAZIDES
29300018701	BISOPROL/HYDROCHLOROTHIAZIDE	THIAZIDES
29300018705	BISOPROL/HYDROCHLOROTHIAZIDE	THIAZIDES
29300018801	BISOPROL/HYDROCHLOROTHIAZIDE	THIAZIDES
29300018805	BISOPROL/HYDROCHLOROTHIAZIDE	THIAZIDES
29300018901	BISOPROL/HYDROCHLOROTHIAZIDE	THIAZIDES
29300018905	BISOPROL/HYDROCHLOROTHIAZIDE	THIAZIDES
29300018913	BISOPROL/HYDROCHLOROTHIAZIDE	THIAZIDES
51285005002	BISOPROL/HYDROCHLOROTHIAZIDE	THIAZIDES
00185070101	BISOPROLOL FUMARATE/HCTZ	THIAZIDES

	GNN	Drug_Class
00185070105	BISOPROLOL FUMARATE/HCTZ	THIAZIDES
00185070130	BISOPROLOL FUMARATE/HCTZ	THIAZIDES
00185070401	BISOPROLOL FUMARATE/HCTZ	THIAZIDES
00185070405	BISOPROLOL FUMARATE/HCTZ	THIAZIDES
00185070430	BISOPROLOL FUMARATE/HCTZ	THIAZIDES
00185070701	BISOPROLOL FUMARATE/HCTZ	THIAZIDES
00185070705	BISOPROLOL FUMARATE/HCTZ	THIAZIDES
00185070730	BISOPROLOL FUMARATE/HCTZ	THIAZIDES
00378050101	BISOPROLOL FUMARATE/HCTZ	THIAZIDES
00378050110	BISOPROLOL FUMARATE/HCTZ	THIAZIDES
00378050301	BISOPROLOL FUMARATE/HCTZ	THIAZIDES
00378050310	BISOPROLOL FUMARATE/HCTZ	THIAZIDES
00378050501	BISOPROLOL FUMARATE/HCTZ	THIAZIDES
00378050505	BISOPROLOL FUMARATE/HCTZ	THIAZIDES
29300018701	BISOPROLOL FUMARATE/HCTZ	THIAZIDES
29300018705	BISOPROLOL FUMARATE/HCTZ	THIAZIDES
29300018713	BISOPROLOL FUMARATE/HCTZ	THIAZIDES
29300018801	BISOPROLOL FUMARATE/HCTZ	THIAZIDES
29300018805	BISOPROLOL FUMARATE/HCTZ	THIAZIDES
29300018813	BISOPROLOL FUMARATE/HCTZ	THIAZIDES
29300018901	BISOPROLOL FUMARATE/HCTZ	THIAZIDES
29300018905	BISOPROLOL FUMARATE/HCTZ	THIAZIDES
29300018913	BISOPROLOL FUMARATE/HCTZ	THIAZIDES
51285004001	BISOPROLOL FUMARATE/HCTZ	THIAZIDES
51285005002	BISOPROLOL FUMARATE/HCTZ	THIAZIDES
00185070101	BISOPROLOL/HYDROCHLOROTHIAZIDE	THIAZIDES
00185070401	BISOPROLOL/HYDROCHLOROTHIAZIDE	THIAZIDES
00185070430	BISOPROLOL/HYDROCHLOROTHIAZIDE	THIAZIDES
00185070701	BISOPROLOL/HYDROCHLOROTHIAZIDE	THIAZIDES
00185070705	BISOPROLOL/HYDROCHLOROTHIAZIDE	THIAZIDES
00185070730	BISOPROLOL/HYDROCHLOROTHIAZIDE	THIAZIDES
00378050101	BISOPROLOL/HYDROCHLOROTHIAZIDE	THIAZIDES
00378050110	BISOPROLOL/HYDROCHLOROTHIAZIDE	THIAZIDES
00378050301	BISOPROLOL/HYDROCHLOROTHIAZIDE	THIAZIDES
00378050310	BISOPROLOL/HYDROCHLOROTHIAZIDE	THIAZIDES
00378050501	BISOPROLOL/HYDROCHLOROTHIAZIDE	THIAZIDES
00378050505	BISOPROLOL/HYDROCHLOROTHIAZIDE	THIAZIDES
29300018701	BISOPROLOL/HYDROCHLOROTHIAZIDE	THIAZIDES
29300018705	BISOPROLOL/HYDROCHLOROTHIAZIDE	THIAZIDES
29300018713	BISOPROLOL/HYDROCHLOROTHIAZIDE	THIAZIDES

© 2024 Hsi RS et al. *JAMA Network Open.*

	GNN	Drug_Class
29300018801	BISOPROLOL/HYDROCHLOROTHIAZIDE	THIAZIDES
29300018805	BISOPROLOL/HYDROCHLOROTHIAZIDE	THIAZIDES
29300018813	BISOPROLOL/HYDROCHLOROTHIAZIDE	THIAZIDES
29300018901	BISOPROLOL/HYDROCHLOROTHIAZIDE	THIAZIDES
29300018905	BISOPROLOL/HYDROCHLOROTHIAZIDE	THIAZIDES
29300018913	BISOPROLOL/HYDROCHLOROTHIAZIDE	THIAZIDES
51285005002	BISOPROLOL/HYDROCHLOROTHIAZIDE	THIAZIDES
00186016254	CANDESARTAN/HYDROCHLOROTHIAZID	THIAZIDES
00186032254	CANDESARTAN/HYDROCHLOROTHIAZID	THIAZIDES
00186032454	CANDESARTAN/HYDROCHLOROTHIAZID	THIAZIDES
00378300105	CANDESARTAN/HYDROCHLOROTHIAZID	THIAZIDES
00378300177	CANDESARTAN/HYDROCHLOROTHIAZID	THIAZIDES
00378300205	CANDESARTAN/HYDROCHLOROTHIAZID	THIAZIDES
00378300277	CANDESARTAN/HYDROCHLOROTHIAZID	THIAZIDES
00378300305	CANDESARTAN/HYDROCHLOROTHIAZID	THIAZIDES
00378300377	CANDESARTAN/HYDROCHLOROTHIAZID	THIAZIDES
33342013110	CANDESARTAN/HYDROCHLOROTHIAZID	THIAZIDES
33342013115	CANDESARTAN/HYDROCHLOROTHIAZID	THIAZIDES
33342013210	CANDESARTAN/HYDROCHLOROTHIAZID	THIAZIDES
33342013310	CANDESARTAN/HYDROCHLOROTHIAZID	THIAZIDES
33342013315	CANDESARTAN/HYDROCHLOROTHIAZID	THIAZIDES
49884066209	CANDESARTAN/HYDROCHLOROTHIAZID	THIAZIDES
49884066309	CANDESARTAN/HYDROCHLOROTHIAZID	THIAZIDES
49884066409	CANDESARTAN/HYDROCHLOROTHIAZID	THIAZIDES
60505375809	CANDESARTAN/HYDROCHLOROTHIAZID	THIAZIDES
60505375909	CANDESARTAN/HYDROCHLOROTHIAZID	THIAZIDES
60505376009	CANDESARTAN/HYDROCHLOROTHIAZID	THIAZIDES
62559066090	CANDESARTAN/HYDROCHLOROTHIAZID	THIAZIDES
62559066190	CANDESARTAN/HYDROCHLOROTHIAZID	THIAZIDES
62559066290	CANDESARTAN/HYDROCHLOROTHIAZID	THIAZIDES
00093017601	CAPTOPRIL/HYDROCHLOROTHIAZIDE	THIAZIDES
00093018201	CAPTOPRIL/HYDROCHLOROTHIAZIDE	THIAZIDES
00378008101	CAPTOPRIL/HYDROCHLOROTHIAZIDE	THIAZIDES
00378008301	CAPTOPRIL/HYDROCHLOROTHIAZIDE	THIAZIDES
00378008401	CAPTOPRIL/HYDROCHLOROTHIAZIDE	THIAZIDES
00378008601	CAPTOPRIL/HYDROCHLOROTHIAZIDE	THIAZIDES
00378021301	CHLORTHALIDONE	THIAZIDES
00378021310	CHLORTHALIDONE	THIAZIDES
00378022201	CHLORTHALIDONE	THIAZIDES
00378022210	CHLORTHALIDONE	THIAZIDES

© 2024 Hsi RS et al. *JAMA Network Open.*

	GNN	Drug_Class
16714080001	CHLORTHALIDONE	THIAZIDES
16714080101	CHLORTHALIDONE	THIAZIDES
43598071901	CHLORTHALIDONE	THIAZIDES
50111036201	CHLORTHALIDONE	THIAZIDES
50111036203	CHLORTHALIDONE	THIAZIDES
50111036301	CHLORTHALIDONE	THIAZIDES
51079005801	CHLORTHALIDONE	THIAZIDES
51079005820	CHLORTHALIDONE	THIAZIDES
57664064888	CHLORTHALIDONE	THIAZIDES
57664064988	CHLORTHALIDONE	THIAZIDES
61570002401	CHLORTHALIDONE	THIAZIDES
64980030301	CHLORTHALIDONE	THIAZIDES
64980030310	CHLORTHALIDONE	THIAZIDES
64980030401	CHLORTHALIDONE	THIAZIDES
75834010901	CHLORTHALIDONE	THIAZIDES
75834010910	CHLORTHALIDONE	THIAZIDES
75834011001	CHLORTHALIDONE	THIAZIDES
75834011010	CHLORTHALIDONE	THIAZIDES
00093104401	ENALAPRIL/HYDROCHLOROTHIAZIDE	THIAZIDES
00093105201	ENALAPRIL/HYDROCHLOROTHIAZIDE	THIAZIDES
00378071201	ENALAPRIL/HYDROCHLOROTHIAZIDE	THIAZIDES
00378072301	ENALAPRIL/HYDROCHLOROTHIAZIDE	THIAZIDES
00713104401	ENALAPRIL/HYDROCHLOROTHIAZIDE	THIAZIDES
00713105201	ENALAPRIL/HYDROCHLOROTHIAZIDE	THIAZIDES
49884068601	ENALAPRIL/HYDROCHLOROTHIAZIDE	THIAZIDES
49884068701	ENALAPRIL/HYDROCHLOROTHIAZIDE	THIAZIDES
51672404501	ENALAPRIL/HYDROCHLOROTHIAZIDE	THIAZIDES
51672404601	ENALAPRIL/HYDROCHLOROTHIAZIDE	THIAZIDES
55111013401	ENALAPRIL/HYDROCHLOROTHIAZIDE	THIAZIDES
60505020801	ENALAPRIL/HYDROCHLOROTHIAZIDE	THIAZIDES
60505020901	ENALAPRIL/HYDROCHLOROTHIAZIDE	THIAZIDES
68682014601	ENALAPRIL/HYDROCHLOROTHIAZIDE	THIAZIDES
00074301511	EPROSARTAN/HYDROCHLOROTHIAZIDE	THIAZIDES
00074302011	EPROSARTAN/HYDROCHLOROTHIAZIDE	THIAZIDES
00185034101	FOSINOPRIL/HYDROCHLOROTHIAZIDE	THIAZIDES
00185034201	FOSINOPRIL/HYDROCHLOROTHIAZIDE	THIAZIDES
23155006001	FOSINOPRIL/HYDROCHLOROTHIAZIDE	THIAZIDES
23155006101	FOSINOPRIL/HYDROCHLOROTHIAZIDE	THIAZIDES
57237002601	FOSINOPRIL/HYDROCHLOROTHIAZIDE	THIAZIDES
57237002701	FOSINOPRIL/HYDROCHLOROTHIAZIDE	THIAZIDES

© 2024 Hsi RS et al. *JAMA Network Open.*

	GNN	Drug_Class
59762525004	FOSINOPRIL/HYDROCHLOROTHIAZIDE	THIAZIDES
59762525101	FOSINOPRIL/HYDROCHLOROTHIAZIDE	THIAZIDES
63304040301	FOSINOPRIL/HYDROCHLOROTHIAZIDE	THIAZIDES
63304040401	FOSINOPRIL/HYDROCHLOROTHIAZIDE	THIAZIDES
65862030801	FOSINOPRIL/HYDROCHLOROTHIAZIDE	THIAZIDES
65862030901	FOSINOPRIL/HYDROCHLOROTHIAZIDE	THIAZIDES
68462055401	FOSINOPRIL/HYDROCHLOROTHIAZIDE	THIAZIDES
68462055501	FOSINOPRIL/HYDROCHLOROTHIAZIDE	THIAZIDES
00093208001	HYDROCHLOROTHIAZIDE	THIAZIDES
00093208010	HYDROCHLOROTHIAZIDE	THIAZIDES
00143125601	HYDROCHLOROTHIAZIDE	THIAZIDES
00143125610	HYDROCHLOROTHIAZIDE	THIAZIDES
00143125651	HYDROCHLOROTHIAZIDE	THIAZIDES
00143125701	HYDROCHLOROTHIAZIDE	THIAZIDES
00143125710	HYDROCHLOROTHIAZIDE	THIAZIDES
00143312501	HYDROCHLOROTHIAZIDE	THIAZIDES
00143312505	HYDROCHLOROTHIAZIDE	THIAZIDES
00172208300	HYDROCHLOROTHIAZIDE	THIAZIDES
00172208310	HYDROCHLOROTHIAZIDE	THIAZIDES
00172208360	HYDROCHLOROTHIAZIDE	THIAZIDES
00172208380	HYDROCHLOROTHIAZIDE	THIAZIDES
00172208910	HYDROCHLOROTHIAZIDE	THIAZIDES
00172208960	HYDROCHLOROTHIAZIDE	THIAZIDES
00172208980	HYDROCHLOROTHIAZIDE	THIAZIDES
00172208985	HYDROCHLOROTHIAZIDE	THIAZIDES
00172487010	HYDROCHLOROTHIAZIDE	THIAZIDES
00172487060	HYDROCHLOROTHIAZIDE	THIAZIDES
00172487080	HYDROCHLOROTHIAZIDE	THIAZIDES
00179006530	HYDROCHLOROTHIAZIDE	THIAZIDES
00182055600	HYDROCHLOROTHIAZIDE	THIAZIDES
00182055689	HYDROCHLOROTHIAZIDE	THIAZIDES
00228222196	HYDROCHLOROTHIAZIDE	THIAZIDES
00228222296	HYDROCHLOROTHIAZIDE	THIAZIDES
00228282011	HYDROCHLOROTHIAZIDE	THIAZIDES
00378081001	HYDROCHLOROTHIAZIDE	THIAZIDES
00378081005	HYDROCHLOROTHIAZIDE	THIAZIDES
00378081093	HYDROCHLOROTHIAZIDE	THIAZIDES
00378360110	HYDROCHLOROTHIAZIDE	THIAZIDES
00527141301	HYDROCHLOROTHIAZIDE	THIAZIDES
00527141310	HYDROCHLOROTHIAZIDE	THIAZIDES

© 2024 Hsi RS et al. *JAMA Network Open.*

	GNN	Drug_Class
00527141401	HYDROCHLOROTHIAZIDE	THIAZIDES
00527141410	HYDROCHLOROTHIAZIDE	THIAZIDES
00527163501	HYDROCHLOROTHIAZIDE	THIAZIDES
00527163510	HYDROCHLOROTHIAZIDE	THIAZIDES
00591034701	HYDROCHLOROTHIAZIDE	THIAZIDES
00591034705	HYDROCHLOROTHIAZIDE	THIAZIDES
00603385521	HYDROCHLOROTHIAZIDE	THIAZIDES
00603385525	HYDROCHLOROTHIAZIDE	THIAZIDES
00603385532	HYDROCHLOROTHIAZIDE	THIAZIDES
00603385593	HYDROCHLOROTHIAZIDE	THIAZIDES
00603385621	HYDROCHLOROTHIAZIDE	THIAZIDES
00603385632	HYDROCHLOROTHIAZIDE	THIAZIDES
00603385634	HYDROCHLOROTHIAZIDE	THIAZIDES
00603385721	HYDROCHLOROTHIAZIDE	THIAZIDES
00603385732	HYDROCHLOROTHIAZIDE	THIAZIDES
10135013410	HYDROCHLOROTHIAZIDE	THIAZIDES
16729018201	HYDROCHLOROTHIAZIDE	THIAZIDES
16729018217	HYDROCHLOROTHIAZIDE	THIAZIDES
16729018301	HYDROCHLOROTHIAZIDE	THIAZIDES
16729018317	HYDROCHLOROTHIAZIDE	THIAZIDES
16729018401	HYDROCHLOROTHIAZIDE	THIAZIDES
16729018417	HYDROCHLOROTHIAZIDE	THIAZIDES
23155000801	HYDROCHLOROTHIAZIDE	THIAZIDES
23155000810	HYDROCHLOROTHIAZIDE	THIAZIDES
23155000818	HYDROCHLOROTHIAZIDE	THIAZIDES
23155000910	HYDROCHLOROTHIAZIDE	THIAZIDES
23155004501	HYDROCHLOROTHIAZIDE	THIAZIDES
23155004505	HYDROCHLOROTHIAZIDE	THIAZIDES
23155004601	HYDROCHLOROTHIAZIDE	THIAZIDES
23155004610	HYDROCHLOROTHIAZIDE	THIAZIDES
23155004701	HYDROCHLOROTHIAZIDE	THIAZIDES
23155004710	HYDROCHLOROTHIAZIDE	THIAZIDES
23155013701	HYDROCHLOROTHIAZIDE	THIAZIDES
23155013801	HYDROCHLOROTHIAZIDE	THIAZIDES
23155013810	HYDROCHLOROTHIAZIDE	THIAZIDES
23155013901	HYDROCHLOROTHIAZIDE	THIAZIDES
23155013910	HYDROCHLOROTHIAZIDE	THIAZIDES
23155014005	HYDROCHLOROTHIAZIDE	THIAZIDES
29300012801	HYDROCHLOROTHIAZIDE	THIAZIDES
29300012810	HYDROCHLOROTHIAZIDE	THIAZIDES

© 2024 Hsi RS et al. *JAMA Network Open.*

	GNN	Drug_Class
29300012901	HYDROCHLOROTHIAZIDE	THIAZIDES
29300012910	HYDROCHLOROTHIAZIDE	THIAZIDES
29300013001	HYDROCHLOROTHIAZIDE	THIAZIDES
29300013005	HYDROCHLOROTHIAZIDE	THIAZIDES
29300013010	HYDROCHLOROTHIAZIDE	THIAZIDES
42543071010	HYDROCHLOROTHIAZIDE	THIAZIDES
43547039711	HYDROCHLOROTHIAZIDE	THIAZIDES
43547039810	HYDROCHLOROTHIAZIDE	THIAZIDES
50111088603	HYDROCHLOROTHIAZIDE	THIAZIDES
50111088703	HYDROCHLOROTHIAZIDE	THIAZIDES
51079077601	HYDROCHLOROTHIAZIDE	THIAZIDES
51079077620	HYDROCHLOROTHIAZIDE	THIAZIDES
52544062201	HYDROCHLOROTHIAZIDE	THIAZIDES
54458087810	HYDROCHLOROTHIAZIDE	THIAZIDES
54458087910	HYDROCHLOROTHIAZIDE	THIAZIDES
54458092910	HYDROCHLOROTHIAZIDE	THIAZIDES
54458093010	HYDROCHLOROTHIAZIDE	THIAZIDES
54458093910	HYDROCHLOROTHIAZIDE	THIAZIDES
54458094010	HYDROCHLOROTHIAZIDE	THIAZIDES
54458094110	HYDROCHLOROTHIAZIDE	THIAZIDES
54458096910	HYDROCHLOROTHIAZIDE	THIAZIDES
54458097010	HYDROCHLOROTHIAZIDE	THIAZIDES
57237000201	HYDROCHLOROTHIAZIDE	THIAZIDES
57237000205	HYDROCHLOROTHIAZIDE	THIAZIDES
57237000299	HYDROCHLOROTHIAZIDE	THIAZIDES
57664019513	HYDROCHLOROTHIAZIDE	THIAZIDES
57664019518	HYDROCHLOROTHIAZIDE	THIAZIDES
57664019588	HYDROCHLOROTHIAZIDE	THIAZIDES
57664042818	HYDROCHLOROTHIAZIDE	THIAZIDES
57664042918	HYDROCHLOROTHIAZIDE	THIAZIDES
57664042988	HYDROCHLOROTHIAZIDE	THIAZIDES
59746012510	HYDROCHLOROTHIAZIDE	THIAZIDES
59746012710	HYDROCHLOROTHIAZIDE	THIAZIDES
59746038206	HYDROCHLOROTHIAZIDE	THIAZIDES
59746038210	HYDROCHLOROTHIAZIDE	THIAZIDES
59762173502	HYDROCHLOROTHIAZIDE	THIAZIDES
59762173602	HYDROCHLOROTHIAZIDE	THIAZIDES
59762173607	HYDROCHLOROTHIAZIDE	THIAZIDES
59762173702	HYDROCHLOROTHIAZIDE	THIAZIDES
59762173707	HYDROCHLOROTHIAZIDE	THIAZIDES

© 2024 Hsi RS et al. *JAMA Network Open.*

	GNN	Drug_Class
60429088910	HYDROCHLOROTHIAZIDE	THIAZIDES
60505264008	HYDROCHLOROTHIAZIDE	THIAZIDES
63739012810	HYDROCHLOROTHIAZIDE	THIAZIDES
64125013001	HYDROCHLOROTHIAZIDE	THIAZIDES
64125013010	HYDROCHLOROTHIAZIDE	THIAZIDES
64125013101	HYDROCHLOROTHIAZIDE	THIAZIDES
64125013110	HYDROCHLOROTHIAZIDE	THIAZIDES
65862011301	HYDROCHLOROTHIAZIDE	THIAZIDES
65862011399	HYDROCHLOROTHIAZIDE	THIAZIDES
65862013301	HYDROCHLOROTHIAZIDE	THIAZIDES
65862013399	HYDROCHLOROTHIAZIDE	THIAZIDES
65862013401	HYDROCHLOROTHIAZIDE	THIAZIDES
65862013499	HYDROCHLOROTHIAZIDE	THIAZIDES
67253082010	HYDROCHLOROTHIAZIDE	THIAZIDES
67253082011	HYDROCHLOROTHIAZIDE	THIAZIDES
67253082110	HYDROCHLOROTHIAZIDE	THIAZIDES
67253082111	HYDROCHLOROTHIAZIDE	THIAZIDES
68084008601	HYDROCHLOROTHIAZIDE	THIAZIDES
68084008611	HYDROCHLOROTHIAZIDE	THIAZIDES
68084039801	HYDROCHLOROTHIAZIDE	THIAZIDES
68084039811	HYDROCHLOROTHIAZIDE	THIAZIDES
68645034154	HYDROCHLOROTHIAZIDE	THIAZIDES
68645051001	HYDROCHLOROTHIAZIDE	THIAZIDES
68645051054	HYDROCHLOROTHIAZIDE	THIAZIDES
68645051070	HYDROCHLOROTHIAZIDE	THIAZIDES
68645051154	HYDROCHLOROTHIAZIDE	THIAZIDES
68645051170	HYDROCHLOROTHIAZIDE	THIAZIDES
69315013001	HYDROCHLOROTHIAZIDE	THIAZIDES
69315013101	HYDROCHLOROTHIAZIDE	THIAZIDES
69315013110	HYDROCHLOROTHIAZIDE	THIAZIDES
69367012204	HYDROCHLOROTHIAZIDE	THIAZIDES
69367012207	HYDROCHLOROTHIAZIDE	THIAZIDES
69367012307	HYDROCHLOROTHIAZIDE	THIAZIDES
00228257111	INDAPAMIDE	THIAZIDES
00228257196	INDAPAMIDE	THIAZIDES
00228259711	INDAPAMIDE	THIAZIDES
00228259796	INDAPAMIDE	THIAZIDES
00378006901	INDAPAMIDE	THIAZIDES
00378006905	INDAPAMIDE	THIAZIDES
00378008001	INDAPAMIDE	THIAZIDES

© 2024 Hsi RS et al. *JAMA Network Open.*

	GNN	Drug_Class
00378008010	INDAPAMIDE	THIAZIDES
00378008077	INDAPAMIDE	THIAZIDES
42291034890	INDAPAMIDE	THIAZIDES
43975021710	INDAPAMIDE	THIAZIDES
43975030410	INDAPAMIDE	THIAZIDES
49884058901	INDAPAMIDE	THIAZIDES
51079086820	INDAPAMIDE	THIAZIDES
62559051001	INDAPAMIDE	THIAZIDES
62559051101	INDAPAMIDE	THIAZIDES
00024585530	IRBESARTAN/HYDROCHLOROTHIAZIDE	THIAZIDES
00024585590	IRBESARTAN/HYDROCHLOROTHIAZIDE	THIAZIDES
00024585630	IRBESARTAN/HYDROCHLOROTHIAZIDE	THIAZIDES
00024585690	IRBESARTAN/HYDROCHLOROTHIAZIDE	THIAZIDES
00054025413	IRBESARTAN/HYDROCHLOROTHIAZIDE	THIAZIDES
00054025422	IRBESARTAN/HYDROCHLOROTHIAZIDE	THIAZIDES
00054025513	IRBESARTAN/HYDROCHLOROTHIAZIDE	THIAZIDES
00054025522	IRBESARTAN/HYDROCHLOROTHIAZIDE	THIAZIDES
00087277531	IRBESARTAN/HYDROCHLOROTHIAZIDE	THIAZIDES
00087277532	IRBESARTAN/HYDROCHLOROTHIAZIDE	THIAZIDES
00087277631	IRBESARTAN/HYDROCHLOROTHIAZIDE	THIAZIDES
00087277632	IRBESARTAN/HYDROCHLOROTHIAZIDE	THIAZIDES
00087278831	IRBESARTAN/HYDROCHLOROTHIAZIDE	THIAZIDES
00087278832	IRBESARTAN/HYDROCHLOROTHIAZIDE	THIAZIDES
00087287531	IRBESARTAN/HYDROCHLOROTHIAZIDE	THIAZIDES
00087287532	IRBESARTAN/HYDROCHLOROTHIAZIDE	THIAZIDES
00087287631	IRBESARTAN/HYDROCHLOROTHIAZIDE	THIAZIDES
00087287632	IRBESARTAN/HYDROCHLOROTHIAZIDE	THIAZIDES
00093723856	IRBESARTAN/HYDROCHLOROTHIAZIDE	THIAZIDES
00093723898	IRBESARTAN/HYDROCHLOROTHIAZIDE	THIAZIDES
00093723956	IRBESARTAN/HYDROCHLOROTHIAZIDE	THIAZIDES
00093723998	IRBESARTAN/HYDROCHLOROTHIAZIDE	THIAZIDES
00093823256	IRBESARTAN/HYDROCHLOROTHIAZIDE	THIAZIDES
00093823298	IRBESARTAN/HYDROCHLOROTHIAZIDE	THIAZIDES
00093823856	IRBESARTAN/HYDROCHLOROTHIAZIDE	THIAZIDES
00093823898	IRBESARTAN/HYDROCHLOROTHIAZIDE	THIAZIDES
00378303393	IRBESARTAN/HYDROCHLOROTHIAZIDE	THIAZIDES
00378303493	IRBESARTAN/HYDROCHLOROTHIAZIDE	THIAZIDES
00603408802	IRBESARTAN/HYDROCHLOROTHIAZIDE	THIAZIDES
00603408816	IRBESARTAN/HYDROCHLOROTHIAZIDE	THIAZIDES
00603408902	IRBESARTAN/HYDROCHLOROTHIAZIDE	THIAZIDES

© 2024 Hsi RS et al. *JAMA Network Open.*

	GNN	Drug_Class
00603408916	IRBESARTAN/HYDROCHLOROTHIAZIDE	THIAZIDES
00955104530	IRBESARTAN/HYDROCHLOROTHIAZIDE	THIAZIDES
00955104590	IRBESARTAN/HYDROCHLOROTHIAZIDE	THIAZIDES
00955104630	IRBESARTAN/HYDROCHLOROTHIAZIDE	THIAZIDES
00955104690	IRBESARTAN/HYDROCHLOROTHIAZIDE	THIAZIDES
33342005707	IRBESARTAN/HYDROCHLOROTHIAZIDE	THIAZIDES
33342005710	IRBESARTAN/HYDROCHLOROTHIAZIDE	THIAZIDES
33342005807	IRBESARTAN/HYDROCHLOROTHIAZIDE	THIAZIDES
33342005810	IRBESARTAN/HYDROCHLOROTHIAZIDE	THIAZIDES
43547033003	IRBESARTAN/HYDROCHLOROTHIAZIDE	THIAZIDES
43547033009	IRBESARTAN/HYDROCHLOROTHIAZIDE	THIAZIDES
43547033103	IRBESARTAN/HYDROCHLOROTHIAZIDE	THIAZIDES
43547033109	IRBESARTAN/HYDROCHLOROTHIAZIDE	THIAZIDES
60505360303	IRBESARTAN/HYDROCHLOROTHIAZIDE	THIAZIDES
60505360309	IRBESARTAN/HYDROCHLOROTHIAZIDE	THIAZIDES
60505360403	IRBESARTAN/HYDROCHLOROTHIAZIDE	THIAZIDES
60505360409	IRBESARTAN/HYDROCHLOROTHIAZIDE	THIAZIDES
62332005190	IRBESARTAN/HYDROCHLOROTHIAZIDE	THIAZIDES
62332005290	IRBESARTAN/HYDROCHLOROTHIAZIDE	THIAZIDES
68180041306	IRBESARTAN/HYDROCHLOROTHIAZIDE	THIAZIDES
68180041309	IRBESARTAN/HYDROCHLOROTHIAZIDE	THIAZIDES
68180041406	IRBESARTAN/HYDROCHLOROTHIAZIDE	THIAZIDES
68180041409	IRBESARTAN/HYDROCHLOROTHIAZIDE	THIAZIDES
68645040570	IRBESARTAN/HYDROCHLOROTHIAZIDE	THIAZIDES
00006014058	LISINOPRIL/HYDROCHLOROTHIAZIDE	THIAZIDES
00093103501	LISINOPRIL/HYDROCHLOROTHIAZIDE	THIAZIDES
00143126201	LISINOPRIL/HYDROCHLOROTHIAZIDE	THIAZIDES
00143126210	LISINOPRIL/HYDROCHLOROTHIAZIDE	THIAZIDES
00143126301	LISINOPRIL/HYDROCHLOROTHIAZIDE	THIAZIDES
00143126310	LISINOPRIL/HYDROCHLOROTHIAZIDE	THIAZIDES
00143126401	LISINOPRIL/HYDROCHLOROTHIAZIDE	THIAZIDES
00143126410	LISINOPRIL/HYDROCHLOROTHIAZIDE	THIAZIDES
00172503210	LISINOPRIL/HYDROCHLOROTHIAZIDE	THIAZIDES
00172503260	LISINOPRIL/HYDROCHLOROTHIAZIDE	THIAZIDES
00172503270	LISINOPRIL/HYDROCHLOROTHIAZIDE	THIAZIDES
00172503300	LISINOPRIL/HYDROCHLOROTHIAZIDE	THIAZIDES
00172503360	LISINOPRIL/HYDROCHLOROTHIAZIDE	THIAZIDES
00172503370	LISINOPRIL/HYDROCHLOROTHIAZIDE	THIAZIDES
00172503410	LISINOPRIL/HYDROCHLOROTHIAZIDE	THIAZIDES
00172503460	LISINOPRIL/HYDROCHLOROTHIAZIDE	THIAZIDES

© 2024 Hsi RS et al. *JAMA Network Open.*

	GNN	Drug_Class
00172503470	LISINOPRIL/HYDROCHLOROTHIAZIDE	THIAZIDES
00185015201	LISINOPRIL/HYDROCHLOROTHIAZIDE	THIAZIDES
00185015210	LISINOPRIL/HYDROCHLOROTHIAZIDE	THIAZIDES
00185017301	LISINOPRIL/HYDROCHLOROTHIAZIDE	THIAZIDES
00185017310	LISINOPRIL/HYDROCHLOROTHIAZIDE	THIAZIDES
00185710001	LISINOPRIL/HYDROCHLOROTHIAZIDE	THIAZIDES
00185710010	LISINOPRIL/HYDROCHLOROTHIAZIDE	THIAZIDES
00310014210	LISINOPRIL/HYDROCHLOROTHIAZIDE	THIAZIDES
00310014211	LISINOPRIL/HYDROCHLOROTHIAZIDE	THIAZIDES
00310014510	LISINOPRIL/HYDROCHLOROTHIAZIDE	THIAZIDES
00310014511	LISINOPRIL/HYDROCHLOROTHIAZIDE	THIAZIDES
00378101201	LISINOPRIL/HYDROCHLOROTHIAZIDE	THIAZIDES
00378201201	LISINOPRIL/HYDROCHLOROTHIAZIDE	THIAZIDES
00378202501	LISINOPRIL/HYDROCHLOROTHIAZIDE	THIAZIDES
00591086001	LISINOPRIL/HYDROCHLOROTHIAZIDE	THIAZIDES
00591086005	LISINOPRIL/HYDROCHLOROTHIAZIDE	THIAZIDES
00591086101	LISINOPRIL/HYDROCHLOROTHIAZIDE	THIAZIDES
00591086105	LISINOPRIL/HYDROCHLOROTHIAZIDE	THIAZIDES
00591086201	LISINOPRIL/HYDROCHLOROTHIAZIDE	THIAZIDES
00591086205	LISINOPRIL/HYDROCHLOROTHIAZIDE	THIAZIDES
42291039001	LISINOPRIL/HYDROCHLOROTHIAZIDE	THIAZIDES
43547042050	LISINOPRIL/HYDROCHLOROTHIAZIDE	THIAZIDES
43547042150	LISINOPRIL/HYDROCHLOROTHIAZIDE	THIAZIDES
43547042250	LISINOPRIL/HYDROCHLOROTHIAZIDE	THIAZIDES
51079069840	LISINOPRIL/HYDROCHLOROTHIAZIDE	THIAZIDES
52427043690	LISINOPRIL/HYDROCHLOROTHIAZIDE	THIAZIDES
52427043790	LISINOPRIL/HYDROCHLOROTHIAZIDE	THIAZIDES
54458088610	LISINOPRIL/HYDROCHLOROTHIAZIDE	THIAZIDES
54458090702	LISINOPRIL/HYDROCHLOROTHIAZIDE	THIAZIDES
54458091002	LISINOPRIL/HYDROCHLOROTHIAZIDE	THIAZIDES
54458099105	LISINOPRIL/HYDROCHLOROTHIAZIDE	THIAZIDES
54458099110	LISINOPRIL/HYDROCHLOROTHIAZIDE	THIAZIDES
54458099210	LISINOPRIL/HYDROCHLOROTHIAZIDE	THIAZIDES
54458099309	LISINOPRIL/HYDROCHLOROTHIAZIDE	THIAZIDES
54868497701	LISINOPRIL/HYDROCHLOROTHIAZIDE	THIAZIDES
59762329303	LISINOPRIL/HYDROCHLOROTHIAZIDE	THIAZIDES
59762329402	LISINOPRIL/HYDROCHLOROTHIAZIDE	THIAZIDES
59762329404	LISINOPRIL/HYDROCHLOROTHIAZIDE	THIAZIDES
59762329502	LISINOPRIL/HYDROCHLOROTHIAZIDE	THIAZIDES
59762329503	LISINOPRIL/HYDROCHLOROTHIAZIDE	THIAZIDES

© 2024 Hsi RS et al. *JAMA Network Open.*

	GNN	Drug_Class
60429004510	LISINOPRIL/HYDROCHLOROTHIAZIDE	THIAZIDES
60505020503	LISINOPRIL/HYDROCHLOROTHIAZIDE	THIAZIDES
60505020603	LISINOPRIL/HYDROCHLOROTHIAZIDE	THIAZIDES
60505020703	LISINOPRIL/HYDROCHLOROTHIAZIDE	THIAZIDES
63304053601	LISINOPRIL/HYDROCHLOROTHIAZIDE	THIAZIDES
63304053605	LISINOPRIL/HYDROCHLOROTHIAZIDE	THIAZIDES
63304053701	LISINOPRIL/HYDROCHLOROTHIAZIDE	THIAZIDES
63304053705	LISINOPRIL/HYDROCHLOROTHIAZIDE	THIAZIDES
63304053801	LISINOPRIL/HYDROCHLOROTHIAZIDE	THIAZIDES
63304053805	LISINOPRIL/HYDROCHLOROTHIAZIDE	THIAZIDES
65862004301	LISINOPRIL/HYDROCHLOROTHIAZIDE	THIAZIDES
65862004305	LISINOPRIL/HYDROCHLOROTHIAZIDE	THIAZIDES
65862004401	LISINOPRIL/HYDROCHLOROTHIAZIDE	THIAZIDES
65862004405	LISINOPRIL/HYDROCHLOROTHIAZIDE	THIAZIDES
65862004501	LISINOPRIL/HYDROCHLOROTHIAZIDE	THIAZIDES
65862004505	LISINOPRIL/HYDROCHLOROTHIAZIDE	THIAZIDES
68180051801	LISINOPRIL/HYDROCHLOROTHIAZIDE	THIAZIDES
68180051802	LISINOPRIL/HYDROCHLOROTHIAZIDE	THIAZIDES
68180051901	LISINOPRIL/HYDROCHLOROTHIAZIDE	THIAZIDES
68180051902	LISINOPRIL/HYDROCHLOROTHIAZIDE	THIAZIDES
68180052001	LISINOPRIL/HYDROCHLOROTHIAZIDE	THIAZIDES
68180052002	LISINOPRIL/HYDROCHLOROTHIAZIDE	THIAZIDES
68645055654	LISINOPRIL/HYDROCHLOROTHIAZIDE	THIAZIDES
68645055754	LISINOPRIL/HYDROCHLOROTHIAZIDE	THIAZIDES
68645055854	LISINOPRIL/HYDROCHLOROTHIAZIDE	THIAZIDES
00006071728	LOSARTAN/HYDROCHLOROTHIAZIDE	THIAZIDES
00006071731	LOSARTAN/HYDROCHLOROTHIAZIDE	THIAZIDES
00006071754	LOSARTAN/HYDROCHLOROTHIAZIDE	THIAZIDES
00006071782	LOSARTAN/HYDROCHLOROTHIAZIDE	THIAZIDES
00006071786	LOSARTAN/HYDROCHLOROTHIAZIDE	THIAZIDES
00006074528	LOSARTAN/HYDROCHLOROTHIAZIDE	THIAZIDES
00006074531	LOSARTAN/HYDROCHLOROTHIAZIDE	THIAZIDES
00006074554	LOSARTAN/HYDROCHLOROTHIAZIDE	THIAZIDES
00006074582	LOSARTAN/HYDROCHLOROTHIAZIDE	THIAZIDES
00006074728	LOSARTAN/HYDROCHLOROTHIAZIDE	THIAZIDES
00006074731	LOSARTAN/HYDROCHLOROTHIAZIDE	THIAZIDES
00006074754	LOSARTAN/HYDROCHLOROTHIAZIDE	THIAZIDES
00006074781	LOSARTAN/HYDROCHLOROTHIAZIDE	THIAZIDES
00006074782	LOSARTAN/HYDROCHLOROTHIAZIDE	THIAZIDES
00054012622	LOSARTAN/HYDROCHLOROTHIAZIDE	THIAZIDES

© 2024 Hsi RS et al. *JAMA Network Open.*

	GNN	Drug_Class
00054012722	LOSARTAN/HYDROCHLOROTHIAZIDE	THIAZIDES
00054027722	LOSARTAN/HYDROCHLOROTHIAZIDE	THIAZIDES
00093736710	LOSARTAN/HYDROCHLOROTHIAZIDE	THIAZIDES
00093736756	LOSARTAN/HYDROCHLOROTHIAZIDE	THIAZIDES
00093736798	LOSARTAN/HYDROCHLOROTHIAZIDE	THIAZIDES
00093736810	LOSARTAN/HYDROCHLOROTHIAZIDE	THIAZIDES
00093736856	LOSARTAN/HYDROCHLOROTHIAZIDE	THIAZIDES
00093736898	LOSARTAN/HYDROCHLOROTHIAZIDE	THIAZIDES
00093736910	LOSARTAN/HYDROCHLOROTHIAZIDE	THIAZIDES
00093736956	LOSARTAN/HYDROCHLOROTHIAZIDE	THIAZIDES
00093736998	LOSARTAN/HYDROCHLOROTHIAZIDE	THIAZIDES
00378141877	LOSARTAN/HYDROCHLOROTHIAZIDE	THIAZIDES
00378141893	LOSARTAN/HYDROCHLOROTHIAZIDE	THIAZIDES
00378141977	LOSARTAN/HYDROCHLOROTHIAZIDE	THIAZIDES
00378142077	LOSARTAN/HYDROCHLOROTHIAZIDE	THIAZIDES
00603422802	LOSARTAN/HYDROCHLOROTHIAZIDE	THIAZIDES
00603422816	LOSARTAN/HYDROCHLOROTHIAZIDE	THIAZIDES
00603422832	LOSARTAN/HYDROCHLOROTHIAZIDE	THIAZIDES
00603422902	LOSARTAN/HYDROCHLOROTHIAZIDE	THIAZIDES
00603422916	LOSARTAN/HYDROCHLOROTHIAZIDE	THIAZIDES
00603422932	LOSARTAN/HYDROCHLOROTHIAZIDE	THIAZIDES
00603423002	LOSARTAN/HYDROCHLOROTHIAZIDE	THIAZIDES
00603423016	LOSARTAN/HYDROCHLOROTHIAZIDE	THIAZIDES
00603423032	LOSARTAN/HYDROCHLOROTHIAZIDE	THIAZIDES
00781520410	LOSARTAN/HYDROCHLOROTHIAZIDE	THIAZIDES
00781520431	LOSARTAN/HYDROCHLOROTHIAZIDE	THIAZIDES
00781520492	LOSARTAN/HYDROCHLOROTHIAZIDE	THIAZIDES
00781520610	LOSARTAN/HYDROCHLOROTHIAZIDE	THIAZIDES
00781520631	LOSARTAN/HYDROCHLOROTHIAZIDE	THIAZIDES
00781520692	LOSARTAN/HYDROCHLOROTHIAZIDE	THIAZIDES
00781520710	LOSARTAN/HYDROCHLOROTHIAZIDE	THIAZIDES
00781520731	LOSARTAN/HYDROCHLOROTHIAZIDE	THIAZIDES
00781520792	LOSARTAN/HYDROCHLOROTHIAZIDE	THIAZIDES
00781581610	LOSARTAN/HYDROCHLOROTHIAZIDE	THIAZIDES
00781581631	LOSARTAN/HYDROCHLOROTHIAZIDE	THIAZIDES
00781581692	LOSARTAN/HYDROCHLOROTHIAZIDE	THIAZIDES
00781581710	LOSARTAN/HYDROCHLOROTHIAZIDE	THIAZIDES
00781581731	LOSARTAN/HYDROCHLOROTHIAZIDE	THIAZIDES
00781581792	LOSARTAN/HYDROCHLOROTHIAZIDE	THIAZIDES
00781581810	LOSARTAN/HYDROCHLOROTHIAZIDE	THIAZIDES

© 2024 Hsi RS et al. *JAMA Network Open.*

	GNN	Drug_Class
00781581831	LOSARTAN/HYDROCHLOROTHIAZIDE	THIAZIDES
00781581892	LOSARTAN/HYDROCHLOROTHIAZIDE	THIAZIDES
13668011610	LOSARTAN/HYDROCHLOROTHIAZIDE	THIAZIDES
13668011630	LOSARTAN/HYDROCHLOROTHIAZIDE	THIAZIDES
13668011690	LOSARTAN/HYDROCHLOROTHIAZIDE	THIAZIDES
13668011710	LOSARTAN/HYDROCHLOROTHIAZIDE	THIAZIDES
13668011730	LOSARTAN/HYDROCHLOROTHIAZIDE	THIAZIDES
13668011790	LOSARTAN/HYDROCHLOROTHIAZIDE	THIAZIDES
13668011810	LOSARTAN/HYDROCHLOROTHIAZIDE	THIAZIDES
13668011830	LOSARTAN/HYDROCHLOROTHIAZIDE	THIAZIDES
13668011890	LOSARTAN/HYDROCHLOROTHIAZIDE	THIAZIDES
16714022401	LOSARTAN/HYDROCHLOROTHIAZIDE	THIAZIDES
16714022402	LOSARTAN/HYDROCHLOROTHIAZIDE	THIAZIDES
16714022501	LOSARTAN/HYDROCHLOROTHIAZIDE	THIAZIDES
16714022502	LOSARTAN/HYDROCHLOROTHIAZIDE	THIAZIDES
16714022504	LOSARTAN/HYDROCHLOROTHIAZIDE	THIAZIDES
16714022601	LOSARTAN/HYDROCHLOROTHIAZIDE	THIAZIDES
16714022602	LOSARTAN/HYDROCHLOROTHIAZIDE	THIAZIDES
16714022604	LOSARTAN/HYDROCHLOROTHIAZIDE	THIAZIDES
33342005010	LOSARTAN/HYDROCHLOROTHIAZIDE	THIAZIDES
33342005110	LOSARTAN/HYDROCHLOROTHIAZIDE	THIAZIDES
33342005210	LOSARTAN/HYDROCHLOROTHIAZIDE	THIAZIDES
43547042309	LOSARTAN/HYDROCHLOROTHIAZIDE	THIAZIDES
43547042311	LOSARTAN/HYDROCHLOROTHIAZIDE	THIAZIDES
43547042409	LOSARTAN/HYDROCHLOROTHIAZIDE	THIAZIDES
52343011790	LOSARTAN/HYDROCHLOROTHIAZIDE	THIAZIDES
52343011890	LOSARTAN/HYDROCHLOROTHIAZIDE	THIAZIDES
57237020790	LOSARTAN/HYDROCHLOROTHIAZIDE	THIAZIDES
57237020799	LOSARTAN/HYDROCHLOROTHIAZIDE	THIAZIDES
57237020890	LOSARTAN/HYDROCHLOROTHIAZIDE	THIAZIDES
57237020899	LOSARTAN/HYDROCHLOROTHIAZIDE	THIAZIDES
57237020990	LOSARTAN/HYDROCHLOROTHIAZIDE	THIAZIDES
57237020999	LOSARTAN/HYDROCHLOROTHIAZIDE	THIAZIDES
59746033730	LOSARTAN/HYDROCHLOROTHIAZIDE	THIAZIDES
59746033790	LOSARTAN/HYDROCHLOROTHIAZIDE	THIAZIDES
59746033830	LOSARTAN/HYDROCHLOROTHIAZIDE	THIAZIDES
59746033890	LOSARTAN/HYDROCHLOROTHIAZIDE	THIAZIDES
59746033990	LOSARTAN/HYDROCHLOROTHIAZIDE	THIAZIDES
59762001101	LOSARTAN/HYDROCHLOROTHIAZIDE	THIAZIDES
59762001201	LOSARTAN/HYDROCHLOROTHIAZIDE	THIAZIDES

© 2024 Hsi RS et al. *JAMA Network Open.*

	GNN	Drug_Class
59762001501	LOSARTAN/HYDROCHLOROTHIAZIDE	THIAZIDES
60505291503	LOSARTAN/HYDROCHLOROTHIAZIDE	THIAZIDES
60505291509	LOSARTAN/HYDROCHLOROTHIAZIDE	THIAZIDES
60505291603	LOSARTAN/HYDROCHLOROTHIAZIDE	THIAZIDES
60505291609	LOSARTAN/HYDROCHLOROTHIAZIDE	THIAZIDES
60505291703	LOSARTAN/HYDROCHLOROTHIAZIDE	THIAZIDES
60505291709	LOSARTAN/HYDROCHLOROTHIAZIDE	THIAZIDES
62332004830	LOSARTAN/HYDROCHLOROTHIAZIDE	THIAZIDES
62332004890	LOSARTAN/HYDROCHLOROTHIAZIDE	THIAZIDES
62332004891	LOSARTAN/HYDROCHLOROTHIAZIDE	THIAZIDES
62332004930	LOSARTAN/HYDROCHLOROTHIAZIDE	THIAZIDES
62332004990	LOSARTAN/HYDROCHLOROTHIAZIDE	THIAZIDES
62332004991	LOSARTAN/HYDROCHLOROTHIAZIDE	THIAZIDES
62332005030	LOSARTAN/HYDROCHLOROTHIAZIDE	THIAZIDES
62332005090	LOSARTAN/HYDROCHLOROTHIAZIDE	THIAZIDES
62332005091	LOSARTAN/HYDROCHLOROTHIAZIDE	THIAZIDES
63739052810	LOSARTAN/HYDROCHLOROTHIAZIDE	THIAZIDES
65862046830	LOSARTAN/HYDROCHLOROTHIAZIDE	THIAZIDES
65862046890	LOSARTAN/HYDROCHLOROTHIAZIDE	THIAZIDES
65862046899	LOSARTAN/HYDROCHLOROTHIAZIDE	THIAZIDES
65862046930	LOSARTAN/HYDROCHLOROTHIAZIDE	THIAZIDES
65862046990	LOSARTAN/HYDROCHLOROTHIAZIDE	THIAZIDES
65862046999	LOSARTAN/HYDROCHLOROTHIAZIDE	THIAZIDES
65862047030	LOSARTAN/HYDROCHLOROTHIAZIDE	THIAZIDES
65862047090	LOSARTAN/HYDROCHLOROTHIAZIDE	THIAZIDES
65862047099	LOSARTAN/HYDROCHLOROTHIAZIDE	THIAZIDES
68180021503	LOSARTAN/HYDROCHLOROTHIAZIDE	THIAZIDES
68180021506	LOSARTAN/HYDROCHLOROTHIAZIDE	THIAZIDES
68180021509	LOSARTAN/HYDROCHLOROTHIAZIDE	THIAZIDES
68180021603	LOSARTAN/HYDROCHLOROTHIAZIDE	THIAZIDES
68180021606	LOSARTAN/HYDROCHLOROTHIAZIDE	THIAZIDES
68180021609	LOSARTAN/HYDROCHLOROTHIAZIDE	THIAZIDES
68180021703	LOSARTAN/HYDROCHLOROTHIAZIDE	THIAZIDES
68180021706	LOSARTAN/HYDROCHLOROTHIAZIDE	THIAZIDES
68180021709	LOSARTAN/HYDROCHLOROTHIAZIDE	THIAZIDES
68382014206	LOSARTAN/HYDROCHLOROTHIAZIDE	THIAZIDES
68382014210	LOSARTAN/HYDROCHLOROTHIAZIDE	THIAZIDES
68382014216	LOSARTAN/HYDROCHLOROTHIAZIDE	THIAZIDES
68382014306	LOSARTAN/HYDROCHLOROTHIAZIDE	THIAZIDES
68382014310	LOSARTAN/HYDROCHLOROTHIAZIDE	THIAZIDES

© 2024 Hsi RS et al. *JAMA Network Open.*

	GNN	Drug_Class
68382014316	LOSARTAN/HYDROCHLOROTHIAZIDE	THIAZIDES
00378071101	METHYLDOPA/HYDROCHLOROTHIAZIDE	THIAZIDES
00310108730	METOPROLOL SUCCINATE/HCTZ	THIAZIDES
00310109530	METOPROLOL SUCCINATE/HCTZ	THIAZIDES
24987008730	METOPROLOL SUCCINATE/HCTZ	THIAZIDES
24987009530	METOPROLOL SUCCINATE/HCTZ	THIAZIDES
00078046005	METOPROLOL/HYDROCHLOROTHIAZIDE	THIAZIDES
00378042401	METOPROLOL/HYDROCHLOROTHIAZIDE	THIAZIDES
00378043401	METOPROLOL/HYDROCHLOROTHIAZIDE	THIAZIDES
00378044501	METOPROLOL/HYDROCHLOROTHIAZIDE	THIAZIDES
00781563001	METOPROLOL/HYDROCHLOROTHIAZIDE	THIAZIDES
00781563101	METOPROLOL/HYDROCHLOROTHIAZIDE	THIAZIDES
13811066910	METOPROLOL/HYDROCHLOROTHIAZIDE	THIAZIDES
13811067010	METOPROLOL/HYDROCHLOROTHIAZIDE	THIAZIDES
16714041702	METOPROLOL/HYDROCHLOROTHIAZIDE	THIAZIDES
16714041802	METOPROLOL/HYDROCHLOROTHIAZIDE	THIAZIDES
62332011531	METOPROLOL/HYDROCHLOROTHIAZIDE	THIAZIDES
62332011631	METOPROLOL/HYDROCHLOROTHIAZIDE	THIAZIDES
62756036888	METOPROLOL/HYDROCHLOROTHIAZIDE	THIAZIDES
62756037088	METOPROLOL/HYDROCHLOROTHIAZIDE	THIAZIDES
00091372001	MOEXIPRIL/HYDROCHLOROTHIAZIDE	THIAZIDES
00093521301	MOEXIPRIL/HYDROCHLOROTHIAZIDE	THIAZIDES
00093521401	MOEXIPRIL/HYDROCHLOROTHIAZIDE	THIAZIDES
00093521501	MOEXIPRIL/HYDROCHLOROTHIAZIDE	THIAZIDES
00574013301	MOEXIPRIL/HYDROCHLOROTHIAZIDE	THIAZIDES
00574013401	MOEXIPRIL/HYDROCHLOROTHIAZIDE	THIAZIDES
00574013501	MOEXIPRIL/HYDROCHLOROTHIAZIDE	THIAZIDES
16252061301	MOEXIPRIL/HYDROCHLOROTHIAZIDE	THIAZIDES
16252061401	MOEXIPRIL/HYDROCHLOROTHIAZIDE	THIAZIDES
68462020501	MOEXIPRIL/HYDROCHLOROTHIAZIDE	THIAZIDES
68462020601	MOEXIPRIL/HYDROCHLOROTHIAZIDE	THIAZIDES
68462020701	MOEXIPRIL/HYDROCHLOROTHIAZIDE	THIAZIDES
65597011430	OLMESARTAN MED/AMLODIPINE/HCTZ	THIAZIDES
65597011490	OLMESARTAN MED/AMLODIPINE/HCTZ	THIAZIDES
65597011530	OLMESARTAN MED/AMLODIPINE/HCTZ	THIAZIDES
65597011630	OLMESARTAN MED/AMLODIPINE/HCTZ	THIAZIDES
65597011690	OLMESARTAN MED/AMLODIPINE/HCTZ	THIAZIDES
65597011730	OLMESARTAN MED/AMLODIPINE/HCTZ	THIAZIDES
65597011790	OLMESARTAN MED/AMLODIPINE/HCTZ	THIAZIDES
65597011830	OLMESARTAN MED/AMLODIPINE/HCTZ	THIAZIDES

© 2024 Hsi RS et al. *JAMA Network Open.*

	GNN	Drug_Class
65597011890	OLMESARTAN MED/AMLODIPINE/HCTZ	THIAZIDES
00093761556	OLMESARTAN/HYDROCHLOROTHIAZIDE	THIAZIDES
00093761598	OLMESARTAN/HYDROCHLOROTHIAZIDE	THIAZIDES
00093761656	OLMESARTAN/HYDROCHLOROTHIAZIDE	THIAZIDES
00093761698	OLMESARTAN/HYDROCHLOROTHIAZIDE	THIAZIDES
00093761756	OLMESARTAN/HYDROCHLOROTHIAZIDE	THIAZIDES
00093761798	OLMESARTAN/HYDROCHLOROTHIAZIDE	THIAZIDES
00378141377	OLMESARTAN/HYDROCHLOROTHIAZIDE	THIAZIDES
00378141393	OLMESARTAN/HYDROCHLOROTHIAZIDE	THIAZIDES
00378142277	OLMESARTAN/HYDROCHLOROTHIAZIDE	THIAZIDES
00378142293	OLMESARTAN/HYDROCHLOROTHIAZIDE	THIAZIDES
00378142577	OLMESARTAN/HYDROCHLOROTHIAZIDE	THIAZIDES
00378142593	OLMESARTAN/HYDROCHLOROTHIAZIDE	THIAZIDES
13668025130	OLMESARTAN/HYDROCHLOROTHIAZIDE	THIAZIDES
13668025190	OLMESARTAN/HYDROCHLOROTHIAZIDE	THIAZIDES
13668025230	OLMESARTAN/HYDROCHLOROTHIAZIDE	THIAZIDES
13668025290	OLMESARTAN/HYDROCHLOROTHIAZIDE	THIAZIDES
13668025330	OLMESARTAN/HYDROCHLOROTHIAZIDE	THIAZIDES
13668025390	OLMESARTAN/HYDROCHLOROTHIAZIDE	THIAZIDES
43547039103	OLMESARTAN/HYDROCHLOROTHIAZIDE	THIAZIDES
43547039109	OLMESARTAN/HYDROCHLOROTHIAZIDE	THIAZIDES
43547039203	OLMESARTAN/HYDROCHLOROTHIAZIDE	THIAZIDES
43547039209	OLMESARTAN/HYDROCHLOROTHIAZIDE	THIAZIDES
43547039303	OLMESARTAN/HYDROCHLOROTHIAZIDE	THIAZIDES
43547039309	OLMESARTAN/HYDROCHLOROTHIAZIDE	THIAZIDES
57664075883	OLMESARTAN/HYDROCHLOROTHIAZIDE	THIAZIDES
57664075899	OLMESARTAN/HYDROCHLOROTHIAZIDE	THIAZIDES
57664075983	OLMESARTAN/HYDROCHLOROTHIAZIDE	THIAZIDES
57664075999	OLMESARTAN/HYDROCHLOROTHIAZIDE	THIAZIDES
57664076083	OLMESARTAN/HYDROCHLOROTHIAZIDE	THIAZIDES
57664076099	OLMESARTAN/HYDROCHLOROTHIAZIDE	THIAZIDES
62332014930	OLMESARTAN/HYDROCHLOROTHIAZIDE	THIAZIDES
62332014990	OLMESARTAN/HYDROCHLOROTHIAZIDE	THIAZIDES
62332015030	OLMESARTAN/HYDROCHLOROTHIAZIDE	THIAZIDES
62332015090	OLMESARTAN/HYDROCHLOROTHIAZIDE	THIAZIDES
62332015130	OLMESARTAN/HYDROCHLOROTHIAZIDE	THIAZIDES
62332015190	OLMESARTAN/HYDROCHLOROTHIAZIDE	THIAZIDES
65597010530	OLMESARTAN/HYDROCHLOROTHIAZIDE	THIAZIDES
65597010590	OLMESARTAN/HYDROCHLOROTHIAZIDE	THIAZIDES
65597010630	OLMESARTAN/HYDROCHLOROTHIAZIDE	THIAZIDES

© 2024 Hsi RS et al. *JAMA Network Open.*

	GNN	Drug_Class
65597010690	OLMESARTAN/HYDROCHLOROTHIAZIDE	THIAZIDES
65597010730	OLMESARTAN/HYDROCHLOROTHIAZIDE	THIAZIDES
65597010790	OLMESARTAN/HYDROCHLOROTHIAZIDE	THIAZIDES
65862077930	OLMESARTAN/HYDROCHLOROTHIAZIDE	THIAZIDES
65862077990	OLMESARTAN/HYDROCHLOROTHIAZIDE	THIAZIDES
65862078030	OLMESARTAN/HYDROCHLOROTHIAZIDE	THIAZIDES
65862078090	OLMESARTAN/HYDROCHLOROTHIAZIDE	THIAZIDES
65862078130	OLMESARTAN/HYDROCHLOROTHIAZIDE	THIAZIDES
65862078190	OLMESARTAN/HYDROCHLOROTHIAZIDE	THIAZIDES
00378034701	PROPRANOLOL/HYDROCHLOROTHIAZID	THIAZIDES
00378073101	PROPRANOLOL/HYDROCHLOROTHIAZID	THIAZIDES
00378054277	QUINAPRIL/HYDROCHLOROTHIAZIDE	THIAZIDES
00378054377	QUINAPRIL/HYDROCHLOROTHIAZIDE	THIAZIDES
00378054477	QUINAPRIL/HYDROCHLOROTHIAZIDE	THIAZIDES
31722037490	QUINAPRIL/HYDROCHLOROTHIAZIDE	THIAZIDES
31722037590	QUINAPRIL/HYDROCHLOROTHIAZIDE	THIAZIDES
31722037690	QUINAPRIL/HYDROCHLOROTHIAZIDE	THIAZIDES
43386071005	QUINAPRIL/HYDROCHLOROTHIAZIDE	THIAZIDES
43386071009	QUINAPRIL/HYDROCHLOROTHIAZIDE	THIAZIDES
43386071105	QUINAPRIL/HYDROCHLOROTHIAZIDE	THIAZIDES
43386071109	QUINAPRIL/HYDROCHLOROTHIAZIDE	THIAZIDES
43386071205	QUINAPRIL/HYDROCHLOROTHIAZIDE	THIAZIDES
43386071209	QUINAPRIL/HYDROCHLOROTHIAZIDE	THIAZIDES
52152024030	QUINAPRIL/HYDROCHLOROTHIAZIDE	THIAZIDES
59762022001	QUINAPRIL/HYDROCHLOROTHIAZIDE	THIAZIDES
59762022201	QUINAPRIL/HYDROCHLOROTHIAZIDE	THIAZIDES
59762022301	QUINAPRIL/HYDROCHLOROTHIAZIDE	THIAZIDES
60505340909	QUINAPRIL/HYDROCHLOROTHIAZIDE	THIAZIDES
60505341005	QUINAPRIL/HYDROCHLOROTHIAZIDE	THIAZIDES
60505341009	QUINAPRIL/HYDROCHLOROTHIAZIDE	THIAZIDES
60505341105	QUINAPRIL/HYDROCHLOROTHIAZIDE	THIAZIDES
60505341109	QUINAPRIL/HYDROCHLOROTHIAZIDE	THIAZIDES
65862016190	QUINAPRIL/HYDROCHLOROTHIAZIDE	THIAZIDES
65862016290	QUINAPRIL/HYDROCHLOROTHIAZIDE	THIAZIDES
65862016390	QUINAPRIL/HYDROCHLOROTHIAZIDE	THIAZIDES
69097082805	QUINAPRIL/HYDROCHLOROTHIAZIDE	THIAZIDES
69097082905	QUINAPRIL/HYDROCHLOROTHIAZIDE	THIAZIDES
69097083105	QUINAPRIL/HYDROCHLOROTHIAZIDE	THIAZIDES
00025101131	SPIRONOLACT/HYDROCHLOROTHIAZID	THIAZIDES
00025102131	SPIRONOLACT/HYDROCHLOROTHIAZID	THIAZIDES

© 2024 Hsi RS et al. *JAMA Network Open.*

	GNN	Drug_Class
00378014101	SPIRONOLACT/HYDROCHLOROTHIAZID	THIAZIDES
00378014105	SPIRONOLACT/HYDROCHLOROTHIAZID	THIAZIDES
00378040301	SPIRONOLACT/HYDROCHLOROTHIAZID	THIAZIDES
00378040305	SPIRONOLACT/HYDROCHLOROTHIAZID	THIAZIDES
53489014401	SPIRONOLACT/HYDROCHLOROTHIAZID	THIAZIDES
53489014405	SPIRONOLACT/HYDROCHLOROTHIAZID	THIAZIDES
53489014410	SPIRONOLACT/HYDROCHLOROTHIAZID	THIAZIDES
59762501401	SPIRONOLACT/HYDROCHLOROTHIAZID	THIAZIDES
00054054518	TELMISARTAN/HYDROCHLOROTHIAZID	THIAZIDES
00054054618	TELMISARTAN/HYDROCHLOROTHIAZID	THIAZIDES
00054054718	TELMISARTAN/HYDROCHLOROTHIAZID	THIAZIDES
00378290193	TELMISARTAN/HYDROCHLOROTHIAZID	THIAZIDES
00378290277	TELMISARTAN/HYDROCHLOROTHIAZID	THIAZIDES
00378290293	TELMISARTAN/HYDROCHLOROTHIAZID	THIAZIDES
00378290393	TELMISARTAN/HYDROCHLOROTHIAZID	THIAZIDES
00597004237	TELMISARTAN/HYDROCHLOROTHIAZID	THIAZIDES
00597004337	TELMISARTAN/HYDROCHLOROTHIAZID	THIAZIDES
00597004437	TELMISARTAN/HYDROCHLOROTHIAZID	THIAZIDES
00603592616	TELMISARTAN/HYDROCHLOROTHIAZID	THIAZIDES
00603592716	TELMISARTAN/HYDROCHLOROTHIAZID	THIAZIDES
00603592816	TELMISARTAN/HYDROCHLOROTHIAZID	THIAZIDES
13668015930	TELMISARTAN/HYDROCHLOROTHIAZID	THIAZIDES
13668015972	TELMISARTAN/HYDROCHLOROTHIAZID	THIAZIDES
13668016030	TELMISARTAN/HYDROCHLOROTHIAZID	THIAZIDES
13668016072	TELMISARTAN/HYDROCHLOROTHIAZID	THIAZIDES
13668016130	TELMISARTAN/HYDROCHLOROTHIAZID	THIAZIDES
13668016172	TELMISARTAN/HYDROCHLOROTHIAZID	THIAZIDES
33342012807	TELMISARTAN/HYDROCHLOROTHIAZID	THIAZIDES
33342012907	TELMISARTAN/HYDROCHLOROTHIAZID	THIAZIDES
33342013007	TELMISARTAN/HYDROCHLOROTHIAZID	THIAZIDES
43547044203	TELMISARTAN/HYDROCHLOROTHIAZID	THIAZIDES
62332020930	TELMISARTAN/HYDROCHLOROTHIAZID	THIAZIDES
62332021030	TELMISARTAN/HYDROCHLOROTHIAZID	THIAZIDES
62332021130	TELMISARTAN/HYDROCHLOROTHIAZID	THIAZIDES
65862097603	TELMISARTAN/HYDROCHLOROTHIAZID	THIAZIDES
65862097703	TELMISARTAN/HYDROCHLOROTHIAZID	THIAZIDES
65862097803	TELMISARTAN/HYDROCHLOROTHIAZID	THIAZIDES
68180019306	TELMISARTAN/HYDROCHLOROTHIAZID	THIAZIDES
68180019309	TELMISARTAN/HYDROCHLOROTHIAZID	THIAZIDES
68180019406	TELMISARTAN/HYDROCHLOROTHIAZID	THIAZIDES

© 2024 Hsi RS et al. *JAMA Network Open.*

	GNN	Drug_Class
68180019409	TELMISARTAN/HYDROCHLOROTHIAZID	THIAZIDES
68180019506	TELMISARTAN/HYDROCHLOROTHIAZID	THIAZIDES
68180019509	TELMISARTAN/HYDROCHLOROTHIAZID	THIAZIDES
00007365022	TRIAMTERENE/HYDROCHLOROTHIAZID	THIAZIDES
00007365030	TRIAMTERENE/HYDROCHLOROTHIAZID	THIAZIDES
00172295060	TRIAMTERENE/HYDROCHLOROTHIAZID	THIAZIDES
00172295080	TRIAMTERENE/HYDROCHLOROTHIAZID	THIAZIDES
00378046001	TRIAMTERENE/HYDROCHLOROTHIAZID	THIAZIDES
00378046401	TRIAMTERENE/HYDROCHLOROTHIAZID	THIAZIDES
00378135201	TRIAMTERENE/HYDROCHLOROTHIAZID	THIAZIDES
00378135205	TRIAMTERENE/HYDROCHLOROTHIAZID	THIAZIDES
00378135501	TRIAMTERENE/HYDROCHLOROTHIAZID	THIAZIDES
00378135505	TRIAMTERENE/HYDROCHLOROTHIAZID	THIAZIDES
00378253701	TRIAMTERENE/HYDROCHLOROTHIAZID	THIAZIDES
00378253710	TRIAMTERENE/HYDROCHLOROTHIAZID	THIAZIDES
00527163201	TRIAMTERENE/HYDROCHLOROTHIAZID	THIAZIDES
00527163210	TRIAMTERENE/HYDROCHLOROTHIAZID	THIAZIDES
00555048802	TRIAMTERENE/HYDROCHLOROTHIAZID	THIAZIDES
00555048805	TRIAMTERENE/HYDROCHLOROTHIAZID	THIAZIDES
00555064304	TRIAMTERENE/HYDROCHLOROTHIAZID	THIAZIDES
00591034801	TRIAMTERENE/HYDROCHLOROTHIAZID	THIAZIDES
00591034805	TRIAMTERENE/HYDROCHLOROTHIAZID	THIAZIDES
00591034810	TRIAMTERENE/HYDROCHLOROTHIAZID	THIAZIDES
00591042401	TRIAMTERENE/HYDROCHLOROTHIAZID	THIAZIDES
00591042405	TRIAMTERENE/HYDROCHLOROTHIAZID	THIAZIDES
00781100801	TRIAMTERENE/HYDROCHLOROTHIAZID	THIAZIDES
00781100805	TRIAMTERENE/HYDROCHLOROTHIAZID	THIAZIDES
00781112301	TRIAMTERENE/HYDROCHLOROTHIAZID	THIAZIDES
00781112305	TRIAMTERENE/HYDROCHLOROTHIAZID	THIAZIDES
00781207401	TRIAMTERENE/HYDROCHLOROTHIAZID	THIAZIDES
00781207410	TRIAMTERENE/HYDROCHLOROTHIAZID	THIAZIDES
00781271501	TRIAMTERENE/HYDROCHLOROTHIAZID	THIAZIDES
00781506701	TRIAMTERENE/HYDROCHLOROTHIAZID	THIAZIDES
00781506705	TRIAMTERENE/HYDROCHLOROTHIAZID	THIAZIDES
00781506801	TRIAMTERENE/HYDROCHLOROTHIAZID	THIAZIDES
00781506805	TRIAMTERENE/HYDROCHLOROTHIAZID	THIAZIDES
10135058110	TRIAMTERENE/HYDROCHLOROTHIAZID	THIAZIDES
50111050501	TRIAMTERENE/HYDROCHLOROTHIAZID	THIAZIDES
50111053401	TRIAMTERENE/HYDROCHLOROTHIAZID	THIAZIDES
50111053402	TRIAMTERENE/HYDROCHLOROTHIAZID	THIAZIDES

© 2024 Hsi RS et al. *JAMA Network Open.*

	GNN	Drug_Class
51079043320	TRIAMTERENE/HYDROCHLOROTHIAZID	THIAZIDES
51079093501	TRIAMTERENE/HYDROCHLOROTHIAZID	THIAZIDES
51079093520	TRIAMTERENE/HYDROCHLOROTHIAZID	THIAZIDES
60505265601	TRIAMTERENE/HYDROCHLOROTHIAZID	THIAZIDES
60505265605	TRIAMTERENE/HYDROCHLOROTHIAZID	THIAZIDES
60505265701	TRIAMTERENE/HYDROCHLOROTHIAZID	THIAZIDES
60505265705	TRIAMTERENE/HYDROCHLOROTHIAZID	THIAZIDES
62794046001	TRIAMTERENE/HYDROCHLOROTHIAZID	THIAZIDES
62794046401	TRIAMTERENE/HYDROCHLOROTHIAZID	THIAZIDES
68001021500	TRIAMTERENE/HYDROCHLOROTHIAZID	THIAZIDES
68001021503	TRIAMTERENE/HYDROCHLOROTHIAZID	THIAZIDES
68001021600	TRIAMTERENE/HYDROCHLOROTHIAZID	THIAZIDES
68001021603	TRIAMTERENE/HYDROCHLOROTHIAZID	THIAZIDES
68001032700	TRIAMTERENE/HYDROCHLOROTHIAZID	THIAZIDES
68001032703	TRIAMTERENE/HYDROCHLOROTHIAZID	THIAZIDES
68001032800	TRIAMTERENE/HYDROCHLOROTHIAZID	THIAZIDES
68001032803	TRIAMTERENE/HYDROCHLOROTHIAZID	THIAZIDES
00078031406	VALSARTAN/HYDROCHLOROTHIAZIDE	THIAZIDES
00078031433	VALSARTAN/HYDROCHLOROTHIAZIDE	THIAZIDES
00078031434	VALSARTAN/HYDROCHLOROTHIAZIDE	THIAZIDES
00078031506	VALSARTAN/HYDROCHLOROTHIAZIDE	THIAZIDES
00078031515	VALSARTAN/HYDROCHLOROTHIAZIDE	THIAZIDES
00078031517	VALSARTAN/HYDROCHLOROTHIAZIDE	THIAZIDES
00078031534	VALSARTAN/HYDROCHLOROTHIAZIDE	THIAZIDES
00078031567	VALSARTAN/HYDROCHLOROTHIAZIDE	THIAZIDES
00078038306	VALSARTAN/HYDROCHLOROTHIAZIDE	THIAZIDES
00078038315	VALSARTAN/HYDROCHLOROTHIAZIDE	THIAZIDES
00078038317	VALSARTAN/HYDROCHLOROTHIAZIDE	THIAZIDES
00078038334	VALSARTAN/HYDROCHLOROTHIAZIDE	THIAZIDES
00078047111	VALSARTAN/HYDROCHLOROTHIAZIDE	THIAZIDES
00078047115	VALSARTAN/HYDROCHLOROTHIAZIDE	THIAZIDES
00078047134	VALSARTAN/HYDROCHLOROTHIAZIDE	THIAZIDES
00078047167	VALSARTAN/HYDROCHLOROTHIAZIDE	THIAZIDES
00078047211	VALSARTAN/HYDROCHLOROTHIAZIDE	THIAZIDES
00078047215	VALSARTAN/HYDROCHLOROTHIAZIDE	THIAZIDES
00078047234	VALSARTAN/HYDROCHLOROTHIAZIDE	THIAZIDES
00078047267	VALSARTAN/HYDROCHLOROTHIAZIDE	THIAZIDES
00378632105	VALSARTAN/HYDROCHLOROTHIAZIDE	THIAZIDES
00378632177	VALSARTAN/HYDROCHLOROTHIAZIDE	THIAZIDES
00378632205	VALSARTAN/HYDROCHLOROTHIAZIDE	THIAZIDES

© 2024 Hsi RS et al. *JAMA Network Open.*

	GNN	Drug_Class
00378632277	VALSARTAN/HYDROCHLOROTHIAZIDE	THIAZIDES
00378632305	VALSARTAN/HYDROCHLOROTHIAZIDE	THIAZIDES
00378632377	VALSARTAN/HYDROCHLOROTHIAZIDE	THIAZIDES
00378632405	VALSARTAN/HYDROCHLOROTHIAZIDE	THIAZIDES
00378632477	VALSARTAN/HYDROCHLOROTHIAZIDE	THIAZIDES
00378632505	VALSARTAN/HYDROCHLOROTHIAZIDE	THIAZIDES
00378632577	VALSARTAN/HYDROCHLOROTHIAZIDE	THIAZIDES
00591231510	VALSARTAN/HYDROCHLOROTHIAZIDE	THIAZIDES
00591231519	VALSARTAN/HYDROCHLOROTHIAZIDE	THIAZIDES
00591231619	VALSARTAN/HYDROCHLOROTHIAZIDE	THIAZIDES
00591231719	VALSARTAN/HYDROCHLOROTHIAZIDE	THIAZIDES
00591231819	VALSARTAN/HYDROCHLOROTHIAZIDE	THIAZIDES
00591231919	VALSARTAN/HYDROCHLOROTHIAZIDE	THIAZIDES
00603634502	VALSARTAN/HYDROCHLOROTHIAZIDE	THIAZIDES
00603634528	VALSARTAN/HYDROCHLOROTHIAZIDE	THIAZIDES
00603634602	VALSARTAN/HYDROCHLOROTHIAZIDE	THIAZIDES
00603634628	VALSARTAN/HYDROCHLOROTHIAZIDE	THIAZIDES
00603634702	VALSARTAN/HYDROCHLOROTHIAZIDE	THIAZIDES
00603634728	VALSARTAN/HYDROCHLOROTHIAZIDE	THIAZIDES
00603634802	VALSARTAN/HYDROCHLOROTHIAZIDE	THIAZIDES
00603634902	VALSARTAN/HYDROCHLOROTHIAZIDE	THIAZIDES
00603634928	VALSARTAN/HYDROCHLOROTHIAZIDE	THIAZIDES
00781594810	VALSARTAN/HYDROCHLOROTHIAZIDE	THIAZIDES
00781594892	VALSARTAN/HYDROCHLOROTHIAZIDE	THIAZIDES
00781594910	VALSARTAN/HYDROCHLOROTHIAZIDE	THIAZIDES
00781594964	VALSARTAN/HYDROCHLOROTHIAZIDE	THIAZIDES
00781594992	VALSARTAN/HYDROCHLOROTHIAZIDE	THIAZIDES
00781595010	VALSARTAN/HYDROCHLOROTHIAZIDE	THIAZIDES
00781595064	VALSARTAN/HYDROCHLOROTHIAZIDE	THIAZIDES
00781595092	VALSARTAN/HYDROCHLOROTHIAZIDE	THIAZIDES
00781595164	VALSARTAN/HYDROCHLOROTHIAZIDE	THIAZIDES
00781595192	VALSARTAN/HYDROCHLOROTHIAZIDE	THIAZIDES
00781595210	VALSARTAN/HYDROCHLOROTHIAZIDE	THIAZIDES
00781595264	VALSARTAN/HYDROCHLOROTHIAZIDE	THIAZIDES
00781595292	VALSARTAN/HYDROCHLOROTHIAZIDE	THIAZIDES
33342007410	VALSARTAN/HYDROCHLOROTHIAZIDE	THIAZIDES
33342007444	VALSARTAN/HYDROCHLOROTHIAZIDE	THIAZIDES
33342007510	VALSARTAN/HYDROCHLOROTHIAZIDE	THIAZIDES
33342007515	VALSARTAN/HYDROCHLOROTHIAZIDE	THIAZIDES
33342007610	VALSARTAN/HYDROCHLOROTHIAZIDE	THIAZIDES

© 2024 Hsi RS et al. *JAMA Network Open.*

	GNN	Drug_Class
33342007615	VALSARTAN/HYDROCHLOROTHIAZIDE	THIAZIDES
33342007710	VALSARTAN/HYDROCHLOROTHIAZIDE	THIAZIDES
33342007715	VALSARTAN/HYDROCHLOROTHIAZIDE	THIAZIDES
33342007810	VALSARTAN/HYDROCHLOROTHIAZIDE	THIAZIDES
33342007815	VALSARTAN/HYDROCHLOROTHIAZIDE	THIAZIDES
42291088490	VALSARTAN/HYDROCHLOROTHIAZIDE	THIAZIDES
43547031109	VALSARTAN/HYDROCHLOROTHIAZIDE	THIAZIDES
43547031209	VALSARTAN/HYDROCHLOROTHIAZIDE	THIAZIDES
43547031309	VALSARTAN/HYDROCHLOROTHIAZIDE	THIAZIDES
43547031409	VALSARTAN/HYDROCHLOROTHIAZIDE	THIAZIDES
43547031509	VALSARTAN/HYDROCHLOROTHIAZIDE	THIAZIDES
51079019303	VALSARTAN/HYDROCHLOROTHIAZIDE	THIAZIDES
51079019403	VALSARTAN/HYDROCHLOROTHIAZIDE	THIAZIDES
60505380609	VALSARTAN/HYDROCHLOROTHIAZIDE	THIAZIDES
60505380709	VALSARTAN/HYDROCHLOROTHIAZIDE	THIAZIDES
60505380809	VALSARTAN/HYDROCHLOROTHIAZIDE	THIAZIDES
60505380909	VALSARTAN/HYDROCHLOROTHIAZIDE	THIAZIDES
60505381009	VALSARTAN/HYDROCHLOROTHIAZIDE	THIAZIDES
62332007971	VALSARTAN/HYDROCHLOROTHIAZIDE	THIAZIDES
62332007990	VALSARTAN/HYDROCHLOROTHIAZIDE	THIAZIDES
62332008071	VALSARTAN/HYDROCHLOROTHIAZIDE	THIAZIDES
62332008090	VALSARTAN/HYDROCHLOROTHIAZIDE	THIAZIDES
62332008171	VALSARTAN/HYDROCHLOROTHIAZIDE	THIAZIDES
62332008190	VALSARTAN/HYDROCHLOROTHIAZIDE	THIAZIDES
62332008271	VALSARTAN/HYDROCHLOROTHIAZIDE	THIAZIDES
62332008290	VALSARTAN/HYDROCHLOROTHIAZIDE	THIAZIDES
62332008371	VALSARTAN/HYDROCHLOROTHIAZIDE	THIAZIDES
62332008390	VALSARTAN/HYDROCHLOROTHIAZIDE	THIAZIDES
65862054790	VALSARTAN/HYDROCHLOROTHIAZIDE	THIAZIDES
65862054799	VALSARTAN/HYDROCHLOROTHIAZIDE	THIAZIDES
65862054890	VALSARTAN/HYDROCHLOROTHIAZIDE	THIAZIDES
65862054899	VALSARTAN/HYDROCHLOROTHIAZIDE	THIAZIDES
65862054990	VALSARTAN/HYDROCHLOROTHIAZIDE	THIAZIDES
65862054999	VALSARTAN/HYDROCHLOROTHIAZIDE	THIAZIDES
65862055005	VALSARTAN/HYDROCHLOROTHIAZIDE	THIAZIDES
65862055090	VALSARTAN/HYDROCHLOROTHIAZIDE	THIAZIDES
65862055105	VALSARTAN/HYDROCHLOROTHIAZIDE	THIAZIDES
65862055190	VALSARTAN/HYDROCHLOROTHIAZIDE	THIAZIDES
68084083425	VALSARTAN/HYDROCHLOROTHIAZIDE	THIAZIDES
68180010102	VALSARTAN/HYDROCHLOROTHIAZIDE	THIAZIDES

© 2024 Hsi RS et al. *JAMA Network Open.*

	GNN	Drug_Class
68180010109	VALSARTAN/HYDROCHLOROTHIAZIDE	THIAZIDES
68180010202	VALSARTAN/HYDROCHLOROTHIAZIDE	THIAZIDES
68180010209	VALSARTAN/HYDROCHLOROTHIAZIDE	THIAZIDES
68180010302	VALSARTAN/HYDROCHLOROTHIAZIDE	THIAZIDES
68180010309	VALSARTAN/HYDROCHLOROTHIAZIDE	THIAZIDES
68180010402	VALSARTAN/HYDROCHLOROTHIAZIDE	THIAZIDES
68180010409	VALSARTAN/HYDROCHLOROTHIAZIDE	THIAZIDES
68180010502	VALSARTAN/HYDROCHLOROTHIAZIDE	THIAZIDES
68180010509	VALSARTAN/HYDROCHLOROTHIAZIDE	THIAZIDES

© 2024 Hsi RS et al. *JAMA Network Open.*

eTable 2. Algorithm for Identifying High-Risk Kidney Stone Patients

Risk Factor	Claims-Based Definition
Chronic UTI	ICD-9 diagnosis 590.x; 595.x; or 599.0<br><br>ICD-10 diagnosis N11.0, N30.20, N30.21, N39.0
Cystinuria	ICD-9 diagnosis 270.0<br><br>ICD-10 diagnosis E72.01
Gout and disorders of purine metabolism	ICD-9 diagnosis 274.xx, 277.2x<br><br>ICD-10 diagnosis M10.xx, M1A.xx, E79.1, E79.8
Sarcoidosis	ICD-9 diagnosis 135.xx<br><br>ICD-10 diagnosis D86.x
Osteoporosis/ pathological fractures	ICD-9 diagnosis V17.81, 733.xx, V82.81, or V13.51<br><br>ICD-10 diagnosis M48.4xXA, M48.50XA, M80.08XA, M81.0, M81.8, M84.3xxx, M84.4xxx, M84.68XA, M85.x, M87.x, M89.x, M94.0, M94.20, M94,8X9, M94.9, S02.91XK, S02.92XK, S12.0xxx-S12.6xxx, S22.9XXK, S32.9XXK, S42.xxxx, S52.90xx, S62.90xx, S72.90xx, S82.00xx, S82.90xx, S92.90xx, S92.91xx, Z13.820, Z82.62, Z87.311

© 2024 Hsi RS et al. *JAMA Network Open.*

Bowel resection or bypass	ICD-9 procedure 45.62, 17.31-17.36, 17.39, 52.7x, 44.32, 44.38, 44.39, 45.61, 41.63, 45.8x, or 45.7x<br><br>ICD-10 procedure 0DBE4ZZ, 0DTH4ZZ, 0DTF4ZZ, 0DTL4ZZ, 0DTG4ZZ, 0DTN4ZZ, 0DBE4ZZ, 0DW04UZ, 0DW08UZ, 0D16479, 0D1647A, 0D164J9, 0D164JA, 0D164K9, 0D164KA, 0D164Z9, 0D164ZA, 0D16079, 0D1607A, 0D160J9, 0D160JA, 0D160K9, 0D160KA, 0D160Z9, 0D160ZA, 0D16879, 0D1687A, 0D168J9, 0D168JA, 0D168K9, 0D168KA, 0D168Z9, 0D168ZA, 0DB80ZZ, 0DB83ZZ, 0DB84ZZ, 0DB87ZZ, 0DB88ZZ, 0DT90ZZ, 0DT94ZZ, 0DT97ZZ, 0DT98ZZ, 0DTA0ZZ, 0DTA4ZZ, 0DTA7ZZ, 0DTA8ZZ, 0DTB0ZZ, 0DTB4ZZ, 0DTB7ZZ, 0DTB8ZZ, 0DBE0ZZ, 0DBE3ZZ, 0DBE7ZZ, 0DBE8ZZ, 0DTH0ZZ, 0DTH7ZZ, 0DTH8ZZ, 0DTF0ZZ, 0DTF7ZZ, 0DTF8ZZ, 0DTK0ZZ, 0DTL0ZZ, 0DTL7ZZ, 0DTL8ZZ, 0DTG0ZZ, 0DTG7ZZ, 0DTG8ZZ, 0DTN0ZZ, 0DTN7ZZ, 0DTN8ZZ, 0DBE0ZZ, 0DBE3ZZ, 0DBE7ZZ, 0DBE8ZZ, 0DTE4ZZ, 0DTE0ZZ, 0DTE7ZZ, 0DTE8ZZ, 0D1607A, 0D160JA, 0D160KA, 0D160ZA, 0DT90ZZ, 0DT90ZZ, 0F190Z3, 0F1G0ZC, 0FTG0ZZ, 0FTG0ZZ<br><br>CPT 43620, 43621, 43622, 43631, 43632, 43622, 43634, 43635, 43644, 43645, 43820, 43775, 43825, 43842, 43843, 43845, 43846, 43847,43848, 44025, 44100, 44110, 44111, 44120, 44121, 44126, 44128, 44130, 44139, 44140, 44141, 44143-44147, 44150, 44151, 44155- 44158, 44160, 44188, 44187, 44202, 44203 ,44204, 44205, 44206, 44207, 44208, 44210, 44211, 44212, 44213, 44310, 44312, 4316, 44320, 44340, 44625, 44626, 45113, 45114, 45119, 45120, 45121, 45123, 45126, 45395, 45397
Bariatric Surgery Status	ICD-9 diagnosis V45.3, V45.86<br><br>ICD-10 diagnosis Z98.84, Z98.0<br><br>CPT 43644, 43645, 43770, 43846, 43847
Inflammatory bowel disease	ICD-9 diagnosis 555.x, 556.xx<br><br>ICD-10 diagnosis K50.x, K51.x
Chronic diarrhea	ICD-9 diagnosis 564.1x, 564.5x, or 564.4x, 558.9, 787.91<br><br>ICD-10 diagnosis K52.2, K52.9, K58.0, K58.2, K91.89, K59.1, R19.7
Intestinal malabsorption	ICD-9 diagnosis 579.xx or 564.2x<br><br>ICD-10 diagnosis K90.0 – K90.4, K90.89, K90.9, K91.1, K91.2
Hyperparathyroidism	ICD-9 diagnosis 252.0x<br><br>ICD-10 diagnosis E21.0 – E21.3

© 2024 Hsi RS et al. *JAMA Network Open.*

Immobilization	ICD-9 diagnosis 344.0x, 344.1x, 343.2x, 741.0x, 741.9x, 655.0x, 780.72, 728.3x, or 344.81<br><br>ICD-10 diagnosis G80.0, G82.20, G82.50- G82.54, G83.5, M62.3, M62.89, O35.0XX0, Q05.0-Q05.8, Q07.01-Q07.03, R53.2
Renal Abnormalities	ICD-9 diagnosis 753.3; 753.0<br><br>ICD-10 diagnosis Q60.2, Q60.5, Q63.x
Medullary sponge	ICD-9 diagnosis 753.17<br><br>ICD-10 diagnosis Q61.5
Solitary Kidney	ICD-9 diagnosis V45.73, 753.0<br><br>ICD-10 diagnosis Q60.x, Z90.5
Cystic fibrosis	ICD-9 diagnosis 277.0x<br><br>ICD-10 diagnosis E84.x, E84

Abbreviations: CPT, *Current Procedural Terminology*; ICD, *International Classification of Diseases, Ninth Revision*; UTI, urinary tract infection

© 2024 Hsi RS et al. *JAMA Network Open.*
