# Supplementary material for: Thiazide Dose, Urine Calcium, and Symptomatic Kidney Stone Events
Source: JAMA Netw Open. 2024 Aug 22;7(8):e2428953. doi: 10.1001/jamanetworkopen.2024.28953 (PMC11342138; doi:10.1001/jamanetworkopen.2024.28953)
Supplement: Supplement 2. — Data Sharing Statement [file jamanetwopen-e2428953-s002.pdf]

Data Sharing Statement

Hsi. Thiazide Dose, Urine Calcium, and Symptomatic Kidney Stone Events. *JAMA Netw Open*. Published August 22, 2024. doi:10.1001/jamanetworkopen.2024.28953

Data
Data available: No
